# Lactate-activated macrophages induced aerobic glycolysis and epithelial-mesenchymal transition in breast cancer by regulation of CCL5-CCR5 axis: a positive metabolic feedback loop

**DOI:** 10.18632/oncotarget.22786

**Published:** 2017-11-30

**Authors:** Sensen Lin, Li Sun, Xiaodan Lyu, Xiongfei Ai, Danyu Du, Nan Su, Hongyang Li, Luyong Zhang, Jun Yu, Shengtao Yuan

**Affiliations:** ^1^ Jiangsu Center for Pharmacodynamics Research and Evaluation, China Pharmaceutical University, Nanjing 210009, PR China; ^2^ Department of Molecular Biology, Jiangsu Cancer Hospital, Nanjing 210009, PR China

**Keywords:** macrophage, lactate, CCL5-CCR5 axis, glycolysis, AMPK

## Abstract

Aberrant energy metabolism is critical for cancer progression. Tumor-associated macrophages (TAMs) can stimulate tumor angiogenesis and enhance cancer metastasis; however, the metabolic interaction between cancer cells and macrophages characterized by lactate shuttles remains unclear. Here, we showed that lactate activated human macrophages to a TAM-like phenotype and stimulated the secretion of CCL5 by activation of Notch signaling in macrophages. Reciprocally, CCL5 increased cell migration, induced cancer cell EMT, and promoted aerobic glycolysis in breast cancer cells, suggesting a positive metabolic feedback loop in the co-culture system. Inhibition of CCR5, the cognate receptor of CCL5, or neutralization of CCL5, broke the metabolic loop and decreased cancer cell migration and EMT. Inhibition of aerobic glycolysis significantly reduced breast cancer cell EMT, indicated that aerobic glycolysis was necessary for the invasive phenotype of cancer cells. We further showed that TGF-β signaling regulated the expression of CCR5 in the co-culture system, and CCL5 induced glycolysis by mediation of AMPK signaling. The expression of CCL5-CCR5 axis was highly associated with macrophage infiltration, TGF-β and p-AMPK in clinical samples. CCL5-CCR5 axis promoted breast cancer metastasis *in vivo*. Our findings suggested a pivotal role of CCL5-CCR5 axis in the metabolic communication between cancer cells and macrophages.

## INTRODUCTION

Aberrant energy metabolism is a hallmark of cancer. Even in the presence of ample oxygen, cancer cells source their energy by a high rate of glycolysis followed by lactic acid fermentation in the cytosol, which is known as aerobic glycolysis or the Warburg effect [1]. Although aerobic glycolysis is a much less efficient producer of ATP compared with oxidative phosphorylation, aerobic glycolysis allows much faster, on-demand, ATP production. Aerobic glycolysis not only provides energy to support the growth of tumor, it is also a source of intermediates for many other metabolic pathways, such as the synthesis of fatty acids and the amino acid alanine [2, 3]. Aerobic glycolysis also helps to create a low pH microenvironment that may confer a proliferation advantage for cancer cells. Lactic acid, an end product of aerobic glycolysis, is secreted into tumor microenvironment to fuel other cancer cells that do not have enough energy supplies [4]. A growing body of evidences also suggested the metabolic communication between cancer cells and stromal cells. For example, lactate produced by cancer-associated fibroblasts can be utilized as energy fuel for oxygenated tumor cells [5, 6]. Understanding the metabolic communication in tumor microenvironment characterized by lactate shuttles is critical to elucidate the heterogeneous biological features of tumor.

Among all the stromal cells that are recruited to the tumor site, macrophages are abundant and present at all stages of tumor progression. In the last decade, the fast evolving field of immunometabolism has provided data on the metabolic profile of tumor-associated macrophages (TAMs). In general, TAMs show an increased aerobic glycolysis [7, 8]. TAMs are also reported to use OXPHOS to generate energy, with decreased glutamine levels. To understand the metabolic interaction between TAMs and cancer cells, we treated human macrophages with lactate and found that lactate activated human macrophages to a tumor-associated macrophage (TAM)-like phenotype. Lactic acid also significantly induced the production of CC chemokine ligand 5 (CCL5) through Notch signaling in TAM-like macrophages.

CCL5, also known as RANTES, plays an active role in recruiting a variety of leukocytes into inflammatory sites. CCL5 is expressed in T lymphocytes, macrophages, platelets, synovial fibroblasts and some types of cancer cells [9]. A variety of human cancers, including breast cancer [10], ovarian cancer [11], Hodgkin's lymphoma [12] and prostate cancer [13], can secret CCL5 or express its receptor, CCR5. The CCL5-CCR5 axis may favor tumor development in multiple ways: acting as growth factors, stimulating angiogenesis, modulating the extracellular matrix, inducing the recruitment of additional stromal and inflammatory cells and taking part in immune evasion mechanisms [14]. Presently, the status of CCL5 in cancer metabolism is unclear. We found that lactate-activated macrophages, in turn, induced aerobic glycolysis in breast cancer cells, which was essential to cancer EMT. We therefore hypothesized that CCL5 played a key role in the interaction between breast cancer cells and TAMs and CCL5 might be associate with cancer EMT and aerobic glycolysis. We also investigated possible mechanisms underlying the metabolic feedback loop and showed that TGF-β signaling regulated the expression of CCR5 and CCL5 enhanced aerobic glycolysis by activation of AMPK.

## RESULTS

### Lactate increased the secretion of CCL5 in human macrophages

**Figure 1:**
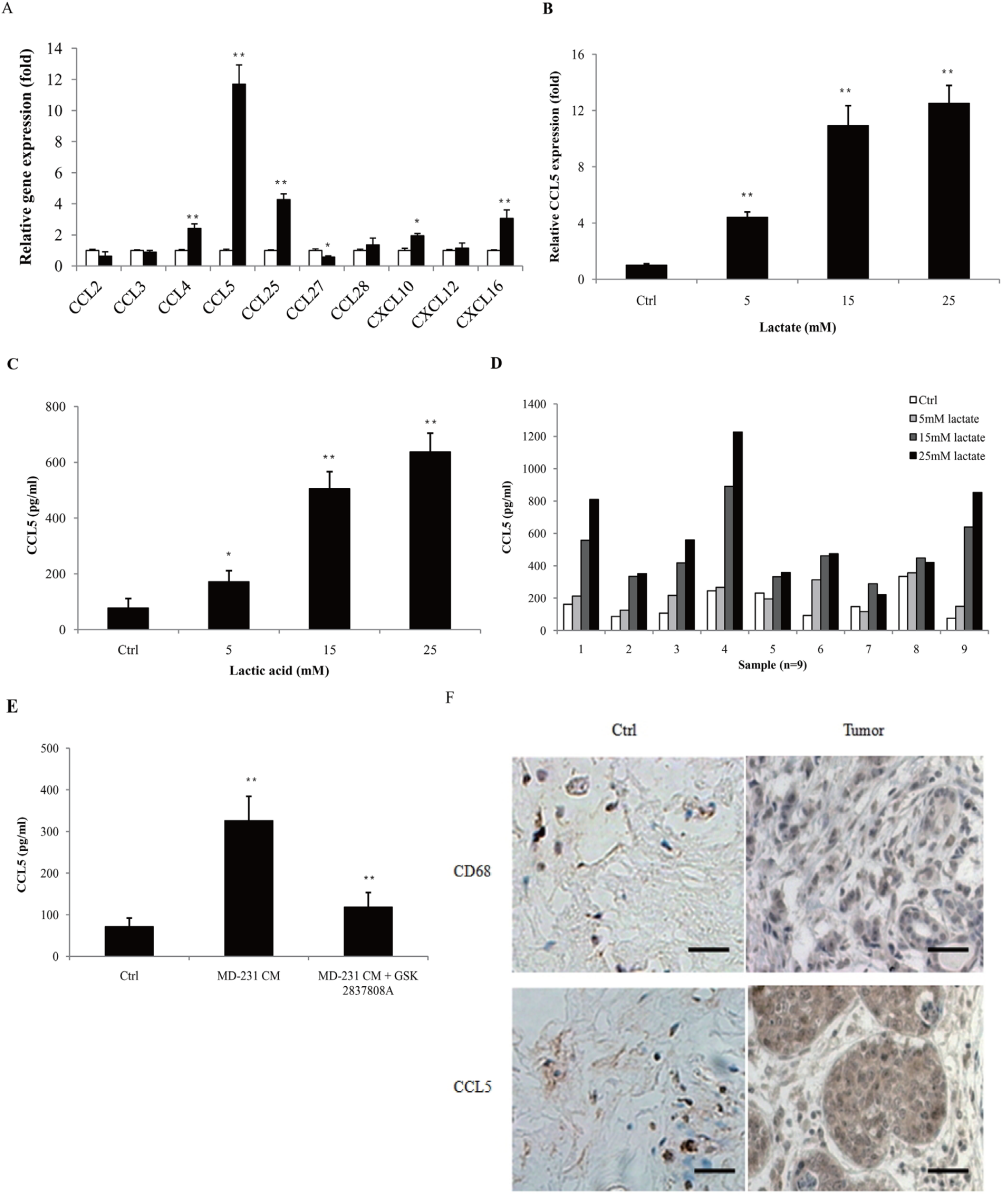
Lactic acid induced the secretion of CCL5 in human macrophages **(A)** 3×10^5^ THP-1 macrophages were treated with 15 mM lactate for 24 h, and the mRNA levels of chemokines were measured by quantitative PCR. The growth medium of control macrophages was titrated to pH6.1 using sterile HCl. **(B)** 3×10^5^ THP-1 macrophages were incubated with different concentrations of lactate for 24 h, and CCL5 gene expression was determined with quantitative PCR. **(C)** 10^6^ THP-1 macrophages were exposed to increasing concentrations of lactate for 48 h, and the secretion of CCL5 was measured by ELISA. **(D)** 10^6^ human primary macrophages from breast cancer patients (n=9) were cultured with different concentrations of lactate for 48 h, and CCL5 production was detected. **(E)** 10^6^ MDA-MB-231 cells were pre-treated with 15μM GSK 2837808A for 2 h, then the media were changed, and cells were cultured for another 24 h. The conditional media (MD-231 CM) were collected and applied to 10^6^ THP-1 macrophages. CCL5 concentrations were detected with ELISA. **(F)** Immunohistochemical staining of CD68 and CCL5 in tumor adjacent tissues (control) and breast tumors (n=28). Scale bars represent 50 μm. ^*^, P<0.05; ^**^, P<0.01.

The concentration of lactic acid in the tumor microenvironment is up to 40 mM [15, 16]. We also showed that human breast cancer cell lines produced large sums of lactic acid (Supplementary Figure 1). To investiagte the effect of lactic acid on human macrophages, we firstly confirmed the polarization of macrophages under the stimulation of lactate. The human monocytic cell line THP-1 was activated by PMA, and the attached cells (THP-1 macrophages) were then treated with 15 mM lactate for 72 h. The gene expression of M2 phenotype marker CD163 and CD206 was significantly up-regulated (Supplementary Figure 2A), while the M1 marker HLA-DRα was decreased (Supplementary Figure 2B). The production of M2 macrophage-associated cytokines, TGF-β1, IL-10 and VEGF, were greatly increased (Supplementary Figure 2C), confirming previous reports that lactate can induce the M2 phenotype in macrophages [17]. Morphologically, lactate-activated THP-1 macrophages showed a honeycomb-like shape and increased intercellular separation (Supplementary Figure 2D). As most TAMs are immature or immunosuppressive (M2 macrophages), these data suggested that lactate induced THP-1 macrophages to a TAM-like phenotype. TAMs contribute to cancer progression by producing a variety of cytokines. To determine the effect of lactate on the secretion of macrophages, THP-1 macrophages were treated with 15 mM lactate for 24 h, and the gene expression of chemokines was investigated by quantitative PCR. As shown in Figure 1A, the expression of CCL4, CCL5, CCL25, CXCL10 and CXCL16 were increased; while the levels of CCL2, CCL3, CCL27, CCL28 and CXCL12 were either not affected or decreased. The gene expression of CCL5 was more potently induced than others, as its mRNA levels accumulated to more than 10-fold higher than the untreated controls. Lactate induced the up-regulation of both CCL5 mRNA (Figure 1B) and CCL5 secretion (Figure 1C) in a dose-dependent manner. The production of other chemokines, such as CCL4, was much less affected (Supplementary Figure 3). We also isolated primary human monocytes from breast cancer patients and differentiated these cells into macrophages. Lactate induced CCL5 secretion in most of the primary macrophages in a dose-dependent manner (Figure 1D). To confirm that cancer cell-derived lactate stimulated CCL5 production, MDA-MB-231 cells were pre-treated with 15μM GSK 2837808A (a selective LDHA inhibitor [18]) for 2 h, and the conditional medium (MD-231 CM) were then collected and applied to THP-1 macrophages. As shown in Figure 1E, MD-231 CM significantly increased the production of CCL5 in THP-1 macrophages; inhibition of LDHA greatly attenuated cancer cell-induced CCL5 secretion. These results clearly showed that cancer cell-derived lactate increased the production of CCL5 in macrophages. We further investigated the status of TAMs and CCL5 in human breast cancer patients (Figure 1F). CD68 is a commonly accepted marker for human macrophages. TAMs were found at all the samples (n=28); compared with adjacent tissues, tumor sections had a higher density of TAM infiltration (Supplementary Figure 4). A significantly higher percentage of CCL5-positive staining was observed in tumors than in matched adjacent tissues, where CCL5 present at low levels (Supplementary Figure 5). As cancer usually prefers to aerobic glycolysis and produces high levels of lactic acid, these results suggested a possible correlation of lactate, TAMs and CCL5 in breast cancer.

### Lactate stimulated the production of CCL5 by activation of Notch signaling in macrophages

**Figure 2:**
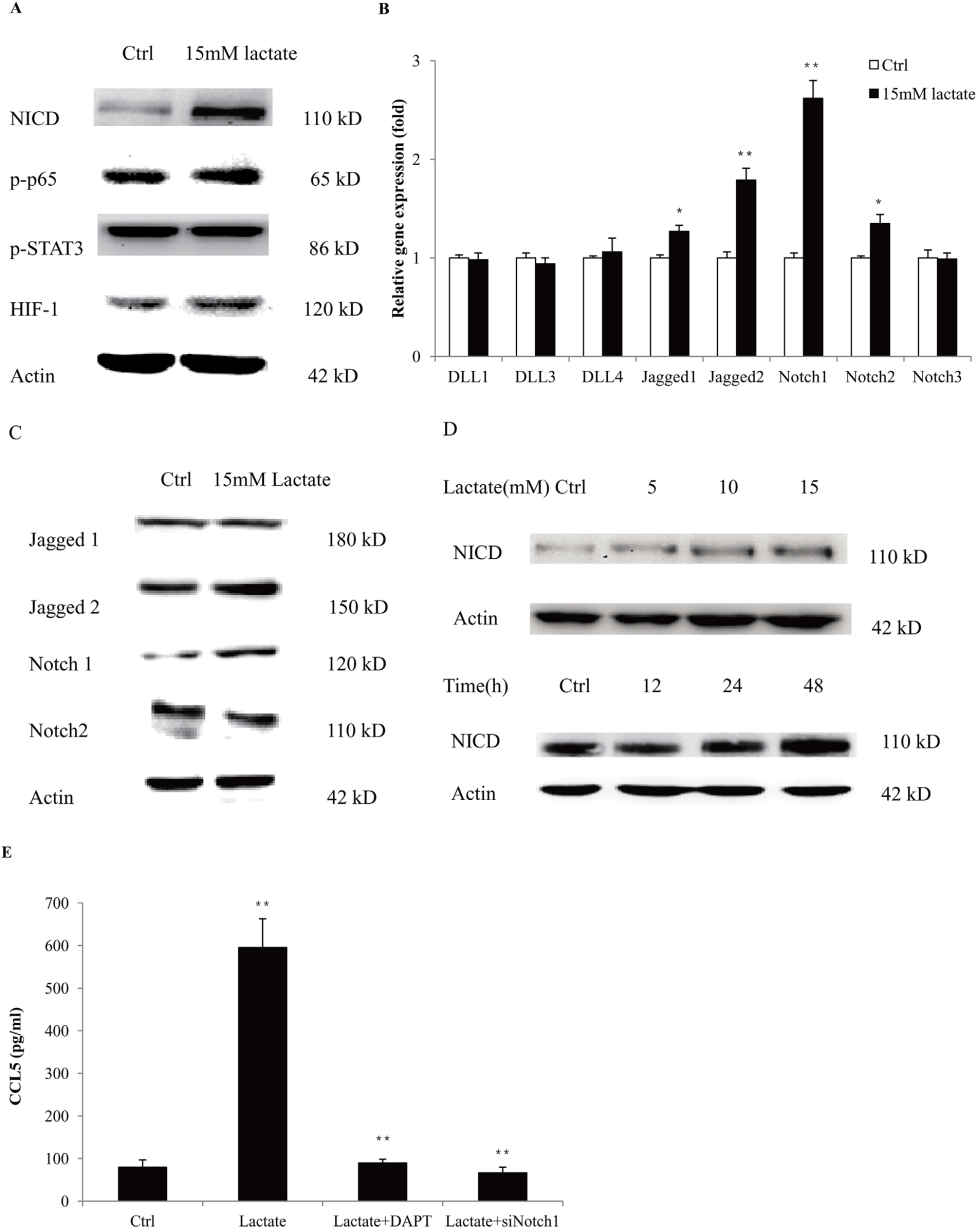
Lactate increased the secretion of CCL5 in macrophages by activation of Notch signaling **(A)** 10^6^ THP-1 macrophages were treated with 15 mM lactate for 48 h, and the expression of key regulators in Notch, NF-κB, STAT3 and HIF, were detected by western blot. **(B)** 3×10^5^ THP-1 macrophages were cultured with 15 mM lactate for 24 h, and the mRNA levels of Notch ligands and receptors were measured by quantitative PCR. **(C)** Western blot for Notch ligands and receptors in THP-1 (10^6^) macrophages after 48 h lactate treatment. **(D)** Lactate stimulated the expression of NICD in a time and dose-dependent manner. 10^6^ THP-1 macrophages were treated with 15 mM lactic acid for 48 h. Data presented were representatives of at least three independent experiments. **(E)** 10^6^ THP-1 macrophages were transfected with 50nM siNotch1, or pretreated with 50μM DAPT for 2 h, and then cultured with 15 mM lactate for 48 h. The secretion of CCL5 was measured by ELISA. ^*^, P<0.05; ^**^, P<0.01.

To investigate the mechanisms of CCL5 secretion in macrophages, we firstly measured the expression of key regulators that are responsible for cytokine production in macrophages. We found that lactate significantly increased the expression of NICD, the Notch intracellular domain, indicating the activation of Notch signaling (Figure 2A). We measured the expressions of Notch receptors and ligands and found that lactate promoted the gene (Figure 2B) and protein expressions (Figure 2C) of Notch1 and Jagged2. Notch signaling is demonstrated to participate in the cell-fate decision of monocytes and the functional modulation of macrophages [19]. We also showed that Notch1/ Jagged2 expressed on the membrane of THP-1 macrophages (Supplementary Figure 6). In fact, lactic acid increased the protein levels of NICD in a time and dose-dependent manner in both THP-1 macrophages (Figure 2D) and primary macrophages (Supplementary Figure 7). To validate the role of Notch signaling in the secretion of CCL5, we inhibited the expression of Notch1 in THP-1 macrophages through RNA interference (Supplementary Figure 8). Silencing of Notch1 significantly reduced the production of CCL5 under lactate stimulation. Furthermore, DAPT, an inhibitor of Notch signaling, also decreased the lactate-induced CCL5 secretion (Figure 2E). These data clearly showed that lactate-stimulated CCL5 production was regulated by Notch signaling in macrophages.

### Lactate-activated macrophages induced breast cancer cell migration and EMT via CCL5-CCR5 axis

**Figure 3:**
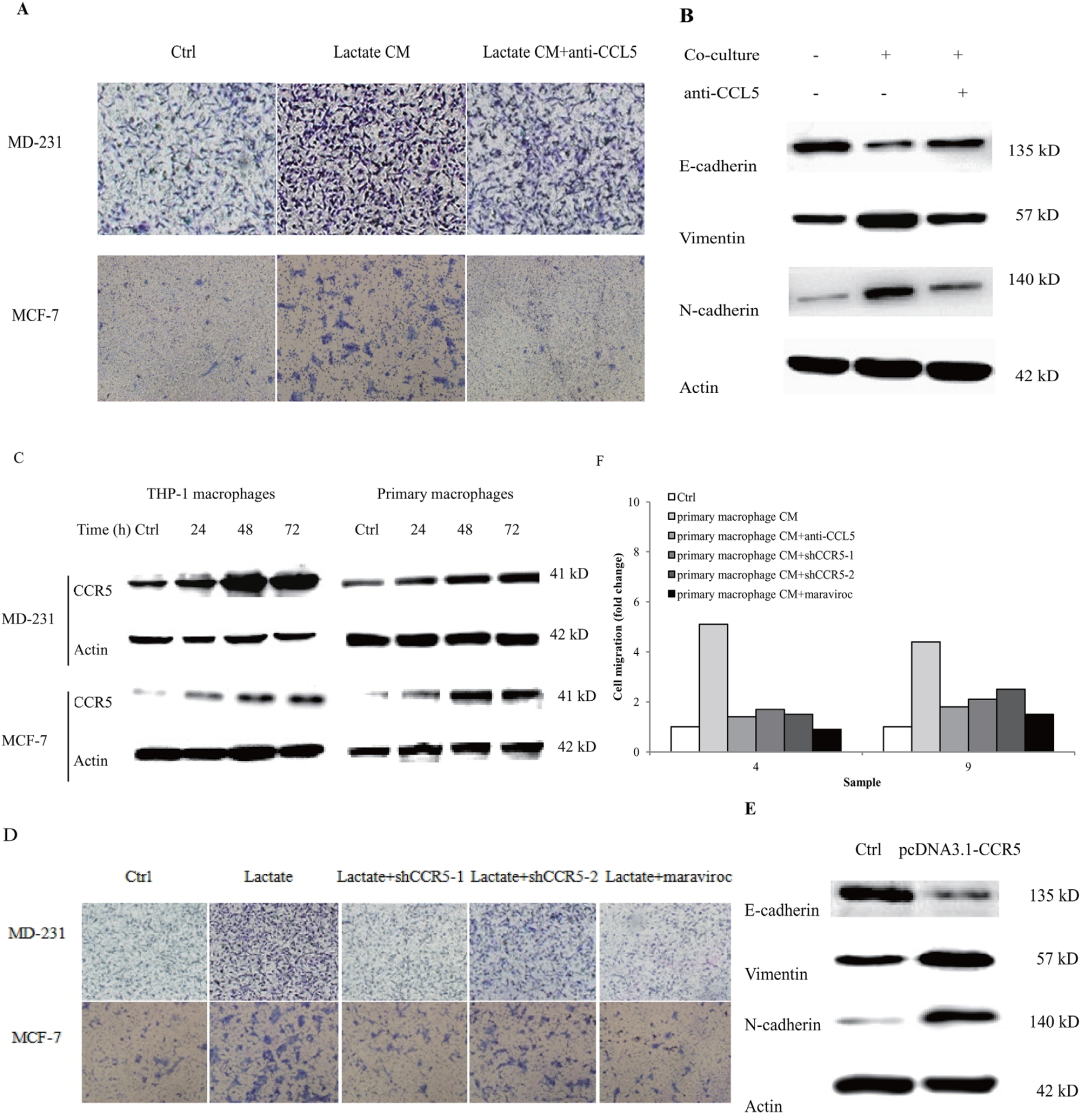
Lactate-activated macrophages induced EMT in breast cancer cells through CCL5-CCR5 axis **(A)** 10^6^ THP-1 macrophages were treated with 15 mM lactate for 72 h, and then cells were washed twice and fresh media were added. Macrophages were cultured for another 24 h and the conditional media (lactate CM) was collected. The effect of CM on breast cancer cell migration was measured by double chamber transwell assay. 5μg/ml anti-CCL5 neutralizing antibody significantly decreased lactate CM-induced cell migration. **(B)** 10^6^ MCF-7 cells were co-cultured with 15 mM lactate-activated macrophages in the presence of 5μg/ml anti-CCL5 antibody or not, and protein levels of EMT markers were tested by western blot. **(C)** 10^6^ breast cancer cells were co-cultured with 10^6^ lactate-activated THP-1 macrophages (or 10^6^ lactate-activated primary macrophages) for different time points, and the expression of CCR5 was monitored by western blot. **(D)** MDA-MB-231 and MCF-7 cells were transfected with shCCR5 plasmids, or pre-treated with 5μM Maraviroc for 2 h, then cell migration induced by lactate CM was detected by double chamber transwell assay. Lactate CM was described in (A). **(E)** MCF-7 cells (10^6^) were transfected with pcDNA3.1-CCR5, and then cultured with 10ng/ml CCL5 for 24 h. The expression of E-cadherin, N-cadherin and vimentin was investigated by western blot. **(F)** 10^6^ Human primary macrophages (No. 4 and No. 9) were treated with 15 mM lactate for 72 h and CM was collected as described in (A). The migration of MDA-MB-231 cells was measured in the presence of primary macrophage CM. 5μg/ml anti-CCL5 neutralizing antibody, shRNAs designed against CCR5, or 5μM Maraviroc, significantly reduced primary macrophage CM-induced cell migration. ^*^, P<0.05; ^**^, P<0.01.

Our previous reports demonstrated that CCL5 contributed significantly to cancer metastasis under hypoxia [20]. Recombinant human CCL5 alone could significantly increase breast cancer cell migration (Supplementary Figure 9). We examined whether lactate-activated macrophages can induce cancer cell migration via CCL5. Breast cancer cells were cultured with the conditional media (CM) collected from 15 mM lactate-activated THP-1 macrophages, and cell migration was measured by the transwell double chamber assay. As shown in Figure 3A, a greater number of cells moved to the lower side of the membrane compared with control cells, indicating that lactate-activated THP-1 macrophages increased breast cancer cell migration. Anti-CCL5 neutralizing antibody significantly inhibited cell migration induced by lactate-activated THP-1 macrophages (Supplementary Figure 10), suggesting that CCL5 was responsible for breast cancer cell migration induced by lactate-activated macrophages.

The most comprehensive mechanism underlying cancer metastasis is the epithelial-mesenchymal transition (EMT). We next assessed the impact of lactate-activated macrophages on breast cancer cell EMT. After co-cultured with lactate-activated THP-1 macrophages for 72 h, most luminal MCF-7 cells lost their epithelial honeycomb-like morphology and obtained a spindle-like shape (Supplementary Figure 11). Along with the morphological changes, the expression of epithelial marker E-cadherin was decreased, whereas the expression of mesenchymal markers (vimentin and N-cadherin) were greatly increased (Figure 3B). Addition of anti-CCL5 neutralizing antibody to the co-culture system inhibited MCF-7 cell EMT. These results indicated that CCL5 was the cytokine responsible for breast cancer EMT induced by lactate-activated THP-1 macrophages.

The cognate receptor of CCL5 is CCR5. We previously reported that breast cancer cells express CCR5 [20]. The surface expression of CCR5 was also confirmed by both flow cytometry and immunofluorescence stanining (Supplementary Figure 12). MDA-MB-231 cells, co-cultured with either lactate-activated THP-1 macrophages, or primary macrophages, showed an increase in CCR5 protein (Figure 3C). MCF-7 cells only showed little expression of CCR5, but co-culture significantly induced CCR5 protein expression in a time-dependent manner. To determine whether macrophage-induced cell migration required CCL5-CCR5 interaction, we inhibited CCR5 expression through shRNA knockdown (Supplementary Figure 13) [20]. Knockdown of CCR5 significantly attenuated the ability of lactate-activated THP-1 macrophages to induce cell migration, as evidenced by the transwell assay. Maraviroc, the inhibitor of CCR5, also prevented breast cancer cell migration induced by macrophages (Figure 3D, Supplementary Figure 14). We next overexpressed CCR5 in breast cancer cells and investigated its effects on EMT. Overexpression of CCR5 (Supplementary Figure 15) in luminal MCF-7 cells that cultured with CCL5, induced EMT (Figure 3E). We also collected the the CM of human primary macrophages after lactate stimulation, and showed that either anti-CCL5 neutralizing antibody or CCR5 inhibition could greatly reduce primary macrophage-induced cell migration (Figure 3F). Taken together, these results indicated a clear effect of CCR5-CCL5 interaction on macrophage-induced cancer cell migration and EMT.

### CCR5 was regulated by TGF-β signaling

**Figure 4:**
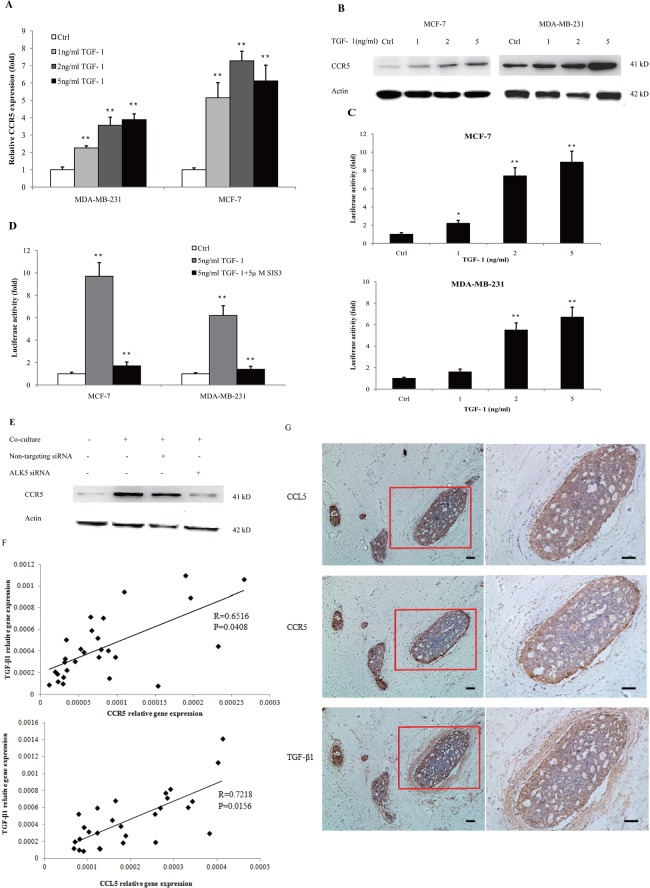
TGF-β signaling regulated the expression of CCR5 **(A)** 3×10^5^ MDA-MB-231 and MCF-7 cells were stimulated with 1-5ng/ml TGF-β1 for 24 h, and total RNA was isolated and tested for CCR5 mRNA by quantitative PCR. **(B)** Western blot for CCR5 protein in breast cancer cells (10^6^) under TGF-β1 stimulation for 48 h. Data presented were representatives of at least three independent experiments. **(C)** MDA-MB-231 and MCF-7 cells (3×10^5^) were co-transfected with pGL3-CCR5 and pRL-TK and exposed to different concentrations of TGF-β1 for 24 h, and luciferase activities were determined. **(D)** MDA-MB-231 and MCF-7 cells were pre-treated with 5μM SIS3 for 2 h, and cells were subjected to luciferase assay. **(E)** 10^6^ MCF-7 cells were transfected with TGFβRI/ALK5 siRNA, and were then co-cultured with lactate-activated THP-1 macrophages (ratio 1:1) for 24 h. The protein levels of CCR5 were assayed by western blot. **(F)** The expression of TGF-β1, CCL5 and CCR5 in clinical samples obtained from breast cancer patients. The mRNA levels were measured by quantitative PCR, and the correlation between TGF-β1 and CCL5-CCR5 axis was shown. **(G)** Representative IHC staining for TGF-β1, CCL5 and CCR5 in breast cancer samples. The sample used was derived from 28 breast cancer cases. Scale bars represent 50 μm. ^*^, P<0.05; ^**^, P<0.01.

Because lactate-activated macrophages produced a large amount of TGF-β (Supplementary Figure 2C), which is an important inducer of EMT, we investigated the relationship between TGF-β signaling and CCL5-CCR5 axis. Previous reports demonstrated the interaction of TGF-β and CCL5 [21]; we also confirmed that TGF-β1 enhanced the the secretion of CCL5 in THP-1 macrophages (Supplementary Figure 16). However, the effect of TGF-β signaling on CCR5 in cancer was not identified yet. As shown in Figure 4A, CCR5 mRNA was significantly increased in MDA-MB-231 and MCF-7 cells that cultured with different concentrations of TGF-β1 for 24 h. TGF-β1 also increased the protein levels of CCR5 in a dose-dependent manner (Figure 4B). These results were confirmed by immunofluorescence staining (Supplementary Figure 17), wherein 5ng/ml TGF-β1 induced a strong increase of CCR5 expression in MCF-7 cells. To investigate the mechanism of CCR5 gene transcription, we performed the dual luciferase reporter gene assay to estimate the activity of CCR5 promoter under TGF-β1 stimulation. MDA-MB-231 and MCF-7 cells were transiently co-transfected with pGL3-CCR5 and pRL-TK and exposed to different concentrations of TGF-β1 for 24 h. We found that TGF-β1 dose-dependently increased the activity of CCR5 promoter (Figure 4C). TGF-β signals were propagated by phosphorylating SMAD proteins, especially SMAD3. To further demonstrate the contribution of SMAD3 on CCR5 regulation, we inhibited SMAD3 by pretreating breast cancer cells with SIS3, a known SMAD3 inhibitor [22], in the luciferase assay. SIS3 treatment nearly abrogated TGFβ1-stimulated luciferase activity (Figure 4D), indicating that the TGF-β/SMAD3 signaling regulated the activity of CCR5 promoter. We also inhibited TGF-β1 signaling by silencing of TGFβRI/ALK5 in MCF-7 cells (Supplementary Figure 18). Knockdown of ALK5 significantly abrogated macrophage-induced CCR5 expression (Figure 4E), indicating that CCR5 was selectively controlled by TGF-β signaling. We finally investigated the clinical relevance of TGF-β and CCL5-CCR5 axis in human breast cancer patients. As shown in Figure 4F, TGF-β1 mRNA levels were highly correlated with CCR5 mRNA (R=0.6516, P=0.0408) and CCL5 mRNA (R=0.7218, P=0.0156) in clinical samples. The immunohistochemical analysis showed that CCR5 was expressed in 20 of 28 tumor sections analyzed, and CCL5 expression was detected in 22 of 28 tumors. TGF-β1 was observed at all the tumor samples analyzed (n=28). Interestingly, CCR5 was highly expressed at the invasive margin of tumors (Figure 4G). The expressions of CCL5 and TGF-β1 at tumor margin were also higher than those at tumor center. Collectively, these data suggested that CCR5 might be selectively regulated by TGF-β signaling.

### Aerobic glycolysis was essential in macrophage-induced EMT

**Figure 5:**
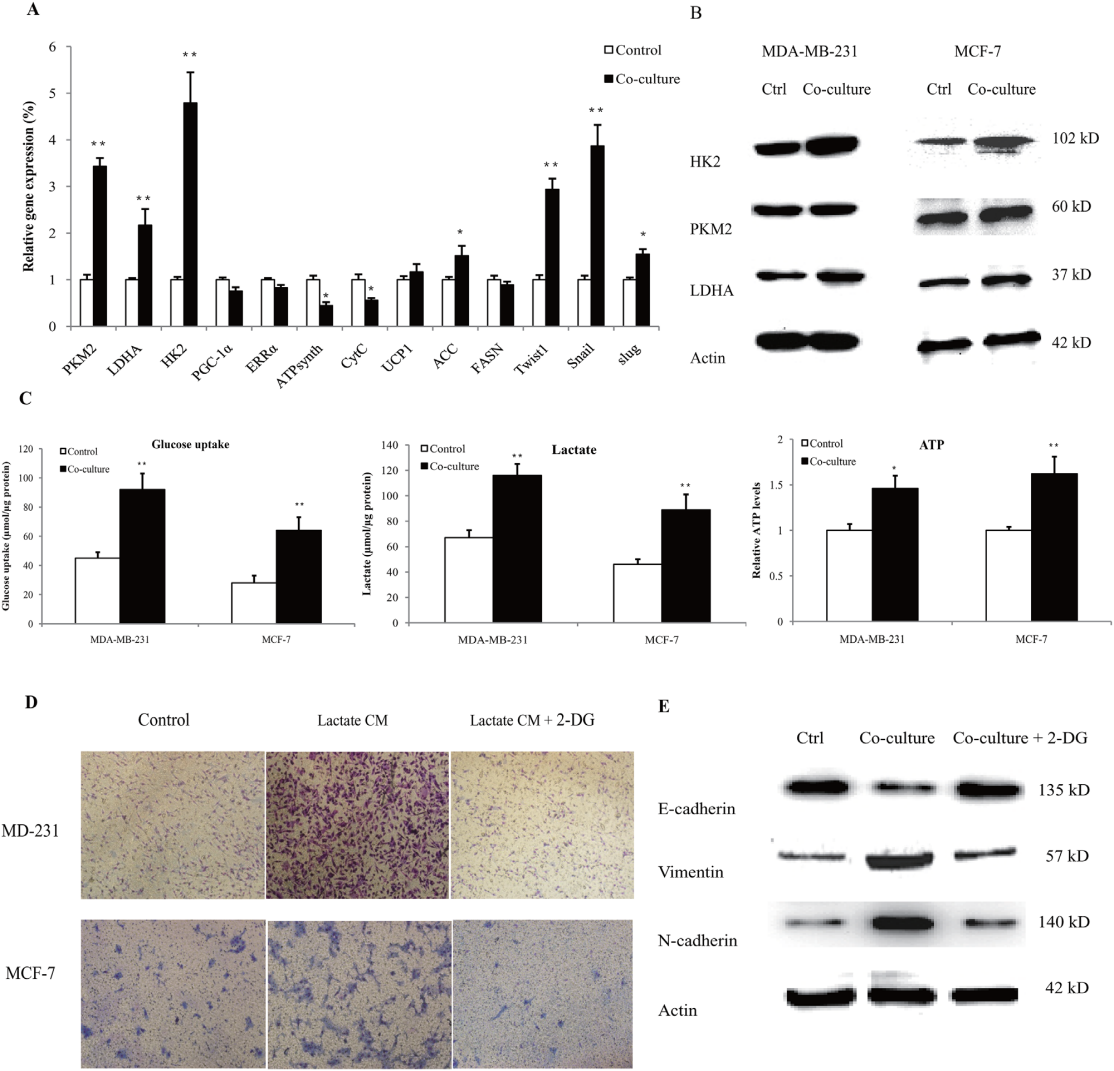
Lactate-activated macrophages induced aerobic glycolysis in breast cancer cells **(A)** 3×10^5^ MDA-MB-231 cells were co-cultured with 15 mM lactate-activated macrophages for 72 h, and the gene expressions of metabolic genes were measured. **(B)** MDA-MB-231 and MCF-7 cells (10^6^) were co-cultured with 15 mM lactate-activated macrophages for 72 h, and the protein levels of glycolytic enzymes were detected by western blot. Data shown were representatives of at least three independent experiments. **(C)** Breast cancer cells were co-cultured with 15 mM lactate-activated macrophages for 72 h. After co-culture, the media were changed with fresh media and cells were further cultured for 24 h. Glucose uptake, lactate production and ATP levels were then detected. **(D)** Breast cancer cells were pre-treated with 10 mM 2-DG for 4 h, and cell migration induced by CM of 15 mM lactate-activated macrophages was detected by double chamber transwell assay. Lactate CM was described in Figure 3A. **(E)** MCF-7 cells were pre-treated with 10 mM 2-DG for 4 h, and then co-cultured with 15 mM lactate-activated macrophages for 72 h. The protein levels of EMT markers were detected by western blot. ^*^, P<0.05; ^**^, P<0.01.

It is intriguing to understand the metabolic interaction in tumor microenvironment. Therefore, we proceeded to explore the effect of human macrophages on cancer cell metabolism. MDA-MB-231 cells were co-cultured with lactate-activated THP-1 macrophages for 72 h, and the expressions of metabolic genes were detected by quantitative PCR. As shown in Figure 5A, genes associated with aerobic glycolysis (HK2, PKM2, LDHA) were significantly up-regulated. We did not observe any significant increase in mitochondrial biogenesis (PGC-1α, ERRα), oxidative phosphorylation (ATPsynth, CytC) and fatty acid metabolism (UCP1, FASN). The mRNA expression of glycolytic genes paralleled that of EMT (Snail, Slug, Twist1), suggesting the utilization of aerobic glycolysis in cancer cells that underwent EMT. The protein levels of glycolytic enzymes were also up-regulated in co-cultured breast cancer cells (Figure 5B). Notably, the protein levels of HK2 in co-cultured MCF-7 cells reached an approximately 3.6-fold higher than control cells (Supplementary Figure 19). We functionally validated our findings by measuring glucose uptake, lactate production and ATP levels. Lactate-activated macrophages, including THP-1 macrophages (Figure 5C) and primary macrophages (Supplementary Figure 20), increased the uptake of glucose, the secretion of lactic acid and ATP levels in both MDA-MB-231 and MCF-7 cells. Treatment with 2-DG, an inhibitor of glycolysis, in breast cancer cells completely abrogated macrophage-induced cancer cell migration and EMT, as evidenced by transwell assay (Figure 5D, Supplementary Figure 21) and the expression profiles of EMT markers (Figure 5E). These results clearly showed a metabolic feedback loop between macrophages and cancer cells, and the macrophage-induced cell EMT was likely caused by aerobic glycolysis.

### CCL5-CCR5 axis promoted aerobic glycolysis by regulation of AMPK signaling

**Figure 6:**
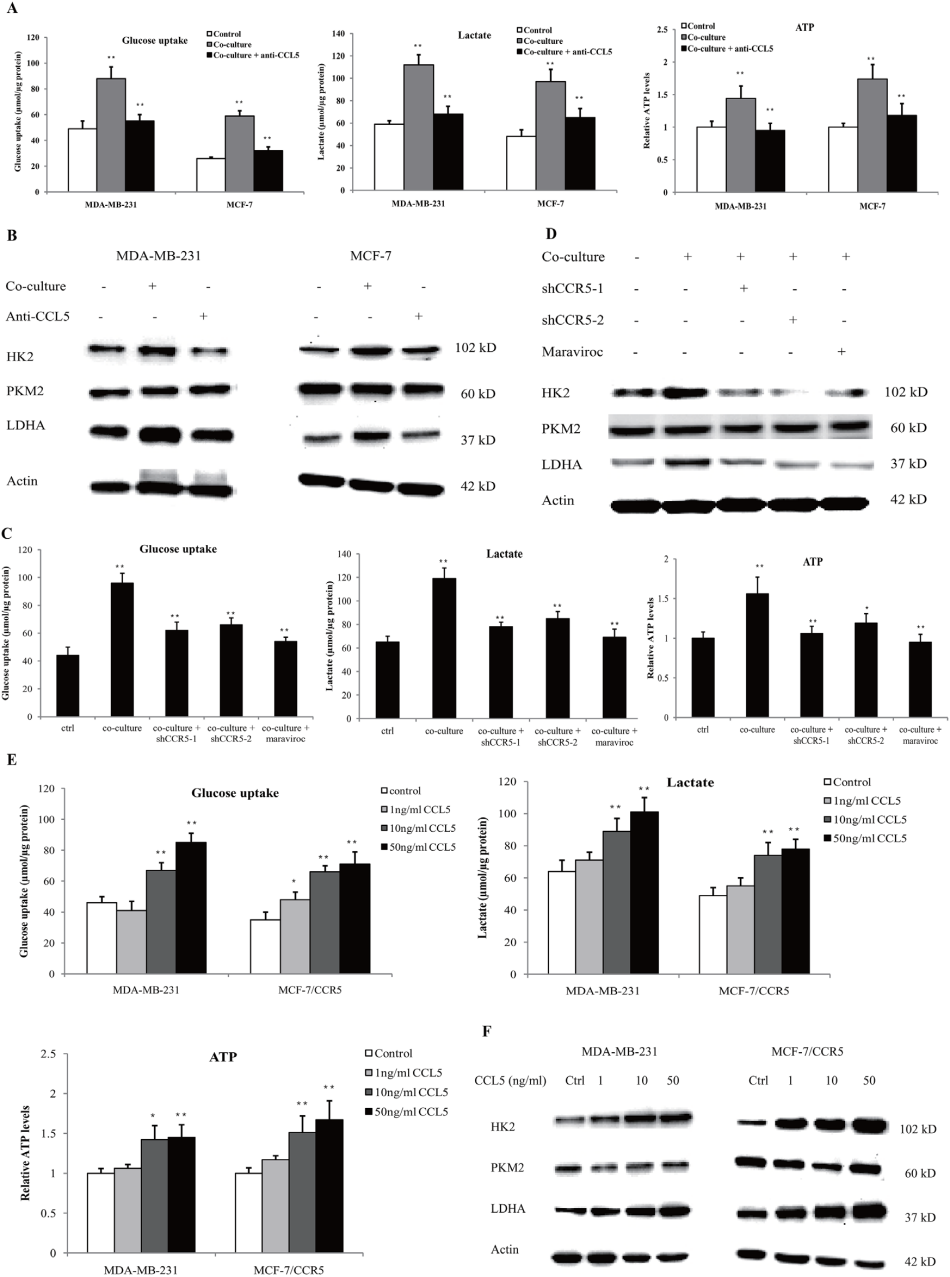
Lactate-activated macrophages induced glycolysis through CCL5-CCR5 axis **(A)** Glucose uptake, lactic acid production and ATP levels in breast cancer cells co-cultured with lactate-activated THP-1 macrophages, with or without 5μg/ml anti-CCL5 neutralizing antibody. The co-culture system was described in Figure 5C. **(B)** Western blots for glycolytic enzymes in breast cancer cells treated as in (A). **(C)** MDA-MB-231 cells were transfected with shRNAs designed against CCR5, or pre-treated with 5μM Maraviroc for 2 h, and then subjected to cell co-culture. Glucose uptake, lactic acid production and ATP levels were measured after co-culture. The co-culture system was described in Figure 5C. **(D)** The protein levels of HK2, PKM2 and LDHA in MDA-MB-231 cells cultured as in (C). **(E)** Recombinant human CCL5 induced aerobic glycolysis in breast cancer cells. MDA-MB-231 and MCF-7/CCR5 cells were treated with increasing concentrations of CCL5 for 12 h, and glucose uptake, lactic acid production and ATP levels were detected. **(F)** Western blots for glycolytic enzymes in MDA-MB-231 and MCF-7/CCR5 cells after stimulation with CCL5. ^*^, P<0.05; ^**^, P<0.01.

To investigate the role of CCL5-CCR5 axis in macrophage-induced cancer aerobic glycolysis, we inhibited CCL5 secretion in the co-culture system using anti-CCL5 neutralizing antibody. As shown in Figure 6A, neutralization of CCL5 significantly attenuated macrophage-induced glucose uptake, lactate secretion and ATP production. Inhibition of CCL5 also reduced the protein expression of glycolytic enzymes in MDA-MB-231 and MCF-7 cells (Figure 6B). Similarly, inhibition of CCR5 in MDA-MB-231 cells abrogated the macrophage-induced glycolysis (Figure 6C), and reduced the expression of glycolytic enzymes in the co-culture system (Figure 6D). Blockade of CCL5-CCR5 axis greatly decreased primary macrophage-induced aerobic glycolysis in MDA-MB-231 cells (Supplementary Figure 22). In addition, recombinant human CCL5 could promote glycolysis (Figure 6E), and enhanced the expression of glycolytic enzymes (Figure 6F) in MDA-MB-231 and MCF-7 cells overexpressing CCR5 (MCF-7/CCR5). TGF-β, which was shown to dictate the expression of CCL5-CCR5 axis in the tumor microenvironment, also induced glycolysis in breast cancer cells (Supplementary Figure 23). Taken together, these results underscored the critical importance of the CCL5–CCR5 axis in macrophage-induced cancer cell glycolysis.

**Figure 7:**
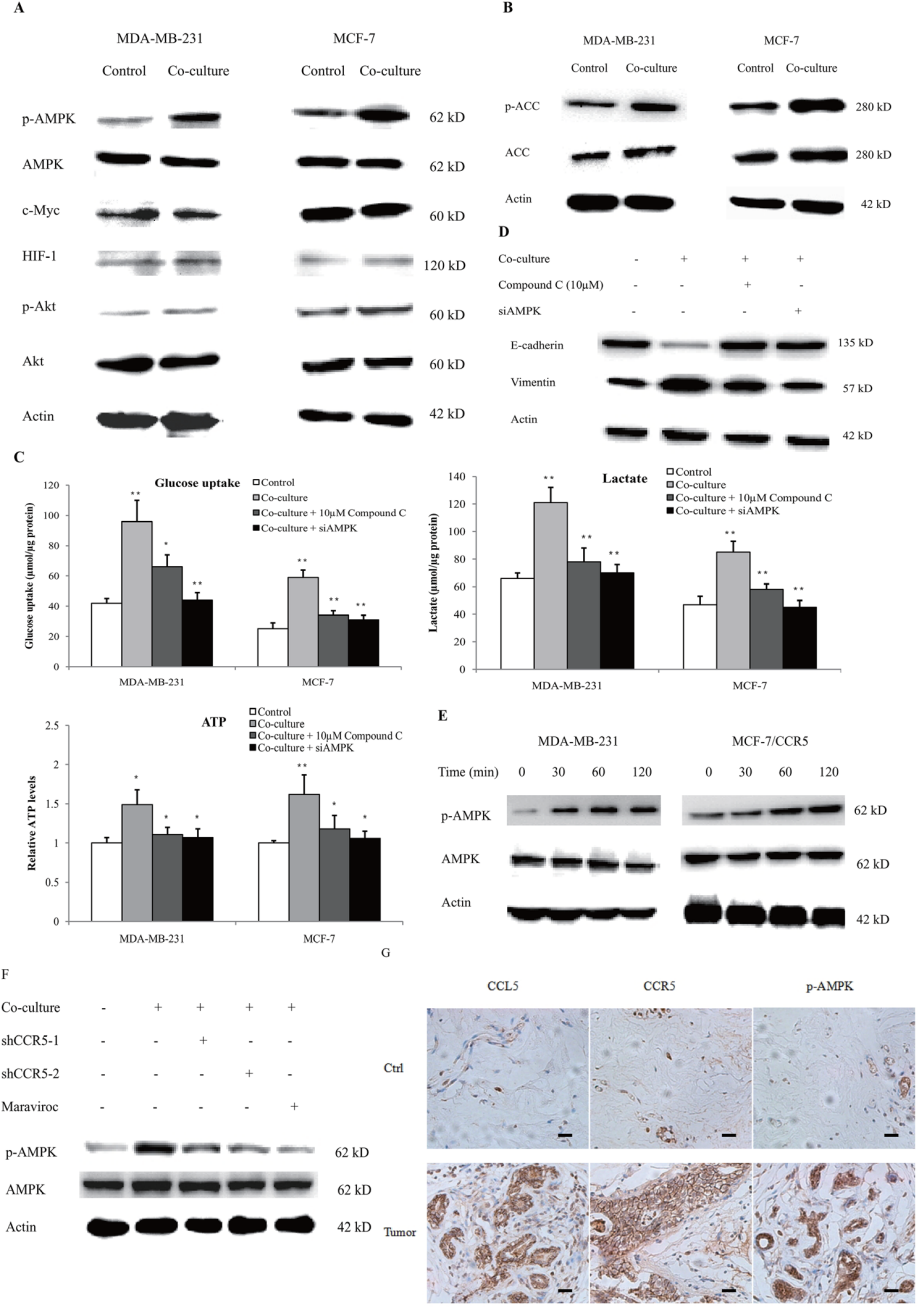
CCL5-CCR5 axis induced aerobic glycolysis by regulation of AMPK signaling **(A)** Western blot for AMPK, c-Myc, HIF-1α and Akt in breast cancer cells co-cultured with 15mM lactic acid-activated THP-1 macrophages (ratio 1:1) for 72 h. Results presented were representatives of at least three independent experiments. **(B)** The expression of AMPK downstream signaling target ACC in breast cancer cells co-cultured as in (A). **(C)** MDA-MB-231 and MCF-7 cells were transfected with 50 nM AMPKα1 siRNA, or pretreated with 10μM compound C for 4 h, and then incubated with 15mM lactic acid-activated THP-1 macrophages (ratio 1:1) for 48 h. The glucose uptake, lactic acid production and ATP levels were detected. **(D)** The inhibition of AMPK abrogated macrophage-induced EMT in MCF-7 cells. Cells were treated as described in (C). After co-culture, the expression of EMT markers, E-cadherin and vimentin, was measured by western blot. **(E)** Recombinant human CCL5 induced the phosphorylation of AMPK in MDA-MB-231 and MCF-7/CCR5 cells. 10^6^ cells were treated with 50ng/ml CCL5 for defferent time points as indicated, and phosphorylated AMPK and total AMPK were investigated by western blot. **(F)** Inhibition of CCR5 in MDA-MB-231 cells significantly attenuated macrophage-induced AMPK phosphorylation. MDA-MB-231 cells were transfected with shRNAs designed against CCR5, or pre-treated with 5μM Maraviroc for 2 h, then co-cultured with 15 mM lactate-activated macrophages as described in (A). After co-culture, the phosphorylation of AMPK was detected by western blot. **(G)** Expressions of CCL5, CCR5 and p-AMPK in samples obtained from breast cancer patients (n =28). Scale bars represent 50 μm. ^*^, P<0.05; ^**^, P<0.01.

Key regulators of glycolysis include HIF-1α, AMPK, PI3K-Akt and c-Myc. To determine which one was mediated by macrophages, we firstly investigated the expression of these regulators in the co-culture system. As shown in Figure 7A, MDA-MB-231 and MCF-7 cells, co-cultured with 15 mM-activated THP-1 macrophages for 48 h, showed significantly increased p-AMPK levels. The phosphorylation of Akt was also slightly up-regulated; however, the expression of HIF-1α and c-Myc was not affected. The phosphorylation of AMPK correlated with the downstream signaling target acetyl-CoA carboxylase (ACC), suggesting the activation of the AMPK-dependent signaling (Figure 7B). The phosphorylation of AMPK in MDA-MB-231 cells that co-cultured with primary macrophages, also significantly up-regulated (Supplementary Figure 24). Inhibition of AMPK by either compound C, the specific inhibitor of AMPK, or AMPK siRNA (Supplementary Figure 25), significantly decreased macrophage-induced aerobic glycolysis (Figure 7C) and EMT (Figure 7D). These data suggested that AMPK signaling was essential to macrophage-induced cancer cell glycolysis and migration. We further explored the linkage between CCL5-CCR5 axis and AMPK and found that CCL5 induced the phosphorylation of AMPK in breast cancer cells (Figure 7E). Inhibition of CCR5 in MDA-MB-231 cells also prohibited the macrophage-induced phosphorylation of AMPK (Figure 7F). We finally validated these findings in breast cancer patients and showed that CCL5-CCR5 axis was significantly correlated with p-AMPK in clinical samples (Figure 7G). Collectively, these results showed that CCL5-CCR5 axis activated AMPK signaling to induce aerobic glycolysis.

### CCL5 increased breast cancer metastasis *in vivo*

**Figure 8:**
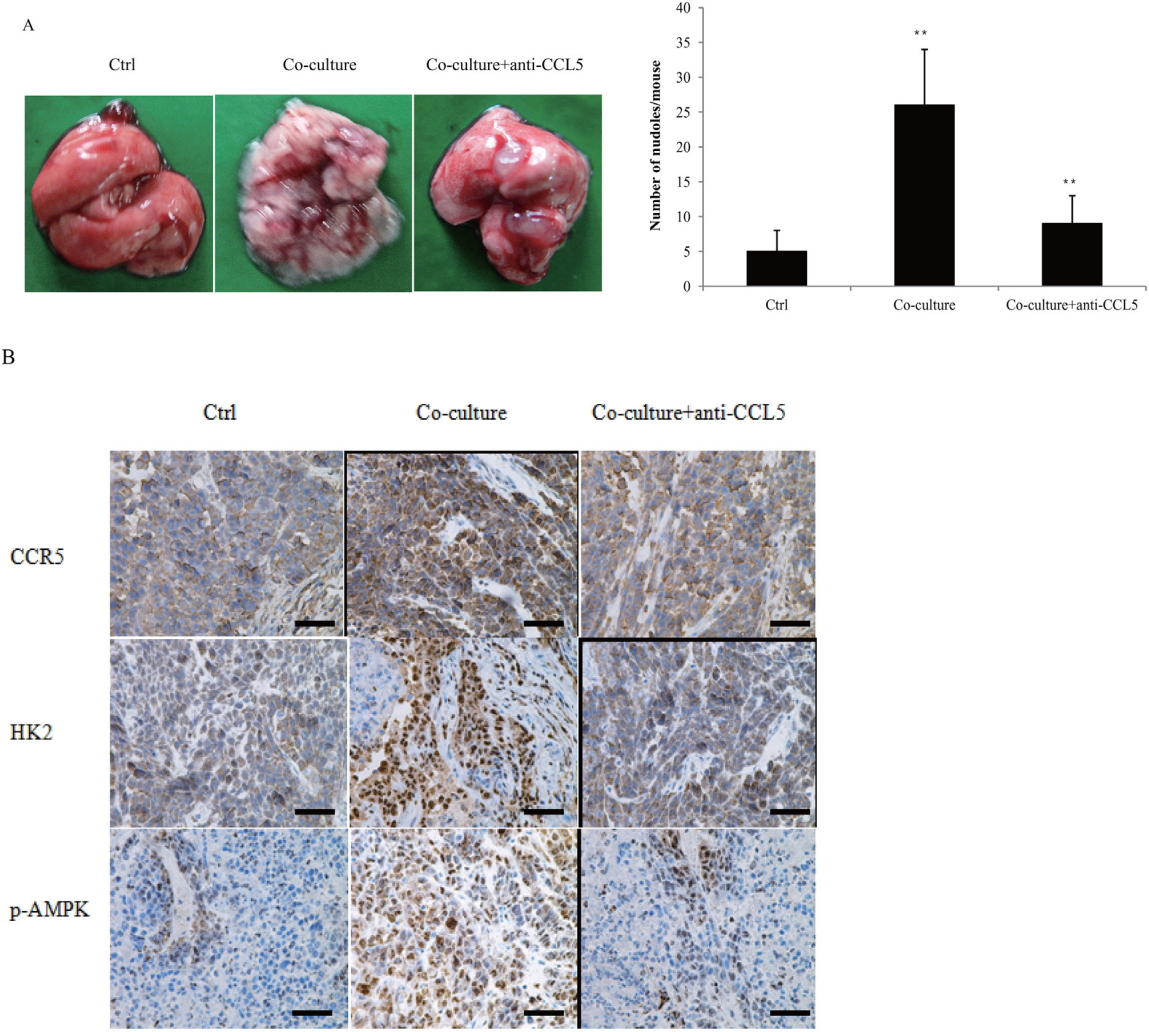
Macrophages promoted breast cancer metastasis through CCL5 **(A)** MDA-MB-231 cells were co-cultured with 15 mM lactate-activated THP-1 macrophages for 7 days, in the presence of 5μg/ml anti-CCL5 neutralizing antibody or not. MDA-MB-231 cells were then collected and injected into the tail vein of nude mice. After two weeks, animals were sacrificed and metastatic nodules on lung surfaces were counted. **(B)** CCR5, HK2 and p-AMPK were immunostained in MDA-MB-231 metastases. Scale bars represent 50 μm. ^*^, P<0.05; ^**^, P<0.01.

We previously reported that CCL5-CCR5 axis promoted breast cancer metastasis under hypoxia [20]. The expression of CCL5-CCR5 axis was correlated with breast cancer lymph node metastasis (Supplementary Tables 1, 2). To validate our findings *in vivo*, MDA-MB-231 cells were co-cultured with 15 mM lactate-activated macrophages for 7 days, and then injected into the tail vein of nude mice. Co-culture significantly promoted breast cancer cell lung metastasis (Figure 8A), and also increased the expression of CCR5, HK2 and p-AMPK in lung metastases (Figure 8B). Addition of anti-CCL5 neutralizing antibody to the co-culture system, not only inhibited breast cancer lung metastasis, but also decreased the expressions of CCR5, HK2 and p-AMPK. These data indicated that CCL5 from macrophages greatly increased breast cancer metastasis.

## DISCUSSION

**Figure 9:**
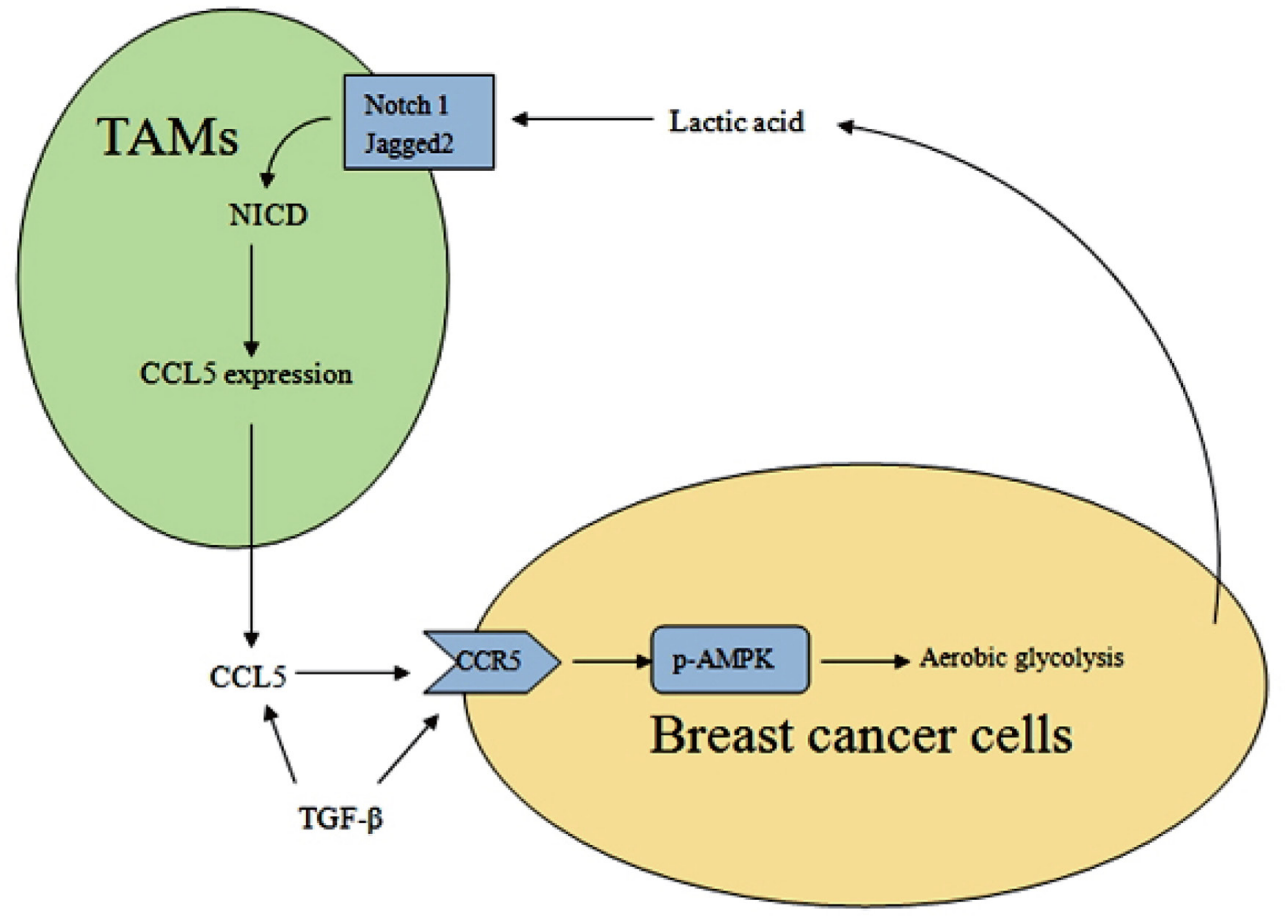
A schematic representation of the metabolic interaction between TAMs and breast cancer cells

As a hallmark of cancer, the metabolism reprogramming in cancer demonstrates the fact that changes in cell metabolism are necessary for tumor initiation and progression. Lactate efflux plays a critical role in tumor-stroma interactions. In this study, we found that cancer cell derived-lactate increased the secretion of CCL5 through Notch signaling in tumor-associated macrophages, and CCL5 in turn induced EMT and aerobic glycolysis in breast cancer cells (Figure 9). We also showed that TGF-β signaling regulated the expression of CCL5-CCR5 axis, which mediated breast cancer aerobic glycolysis by activation of AMPK signaling.

During the preparation of this article, Dr. Gao et al. reported that by regulation of mTOR, CCL5 induced breast cancer metabolic events, specifically glycolysis, to promote cancer proliferation and invasion [23]. In contrast, although we found that CCL5-CCR5 axis played an essential role in the metabolic communication between macrophages and breast cancer cells, we did not observed the activation of mTOR signaling in the co-culture system (data not shown). The mechanisms of the difference are unclear; however, as we used a co-culture system rather than direct stimulation with CCL5, it is possible that macrophages produced many kinds of cytokines, so we observed the “net effects” on downstream signalings.

It is intriguing that CCL5 and CCR5 were reported to be conversely related with TGFβRII in human lung cancer cells [24]. TGFβRII is a core component of TGF-β signaling; however, here we showed that TGF-β increased the expression of CCL5-CCR5 axis. The discrepancy reflected the perturbations of TGF-β signaling in cancer progression because TGF-β signaling is usually altered to circumvent the suppressive effects of the cytokine [25]. In fact, TGFβRII is commonly inactivated in many types of cancer including colorectal, ovarian, lung and head and neck cancers. Beheading of the canonical TGF-β pathway by TGFβRII inactivation can eliminate the suppressive effect of the cytokine, and CCL5 secretion induced by TGFβRII inhibition reflects the non-canonical signaling pathways after TGF-β stimulation. MDA-MB-231 and MCF-7 breast cancer cells harbor the intact TGFβ-SMAD pathway; both our results and previous reports showed that CCL5-CCR5 axis could be selectively mediated by pathological or defective forms of TGF-β signaling.

Interestingly, Notch signaling was reported to induce M1 macrophages and inhibit tumor growth via anti-tumor immune responses [26, 27]. However, conflicting data also presented that Notch signaling can also regulate M2 phenotype in macrophages [28, 29]. These researches suggested that Notch signaling might be required for initial activation and participate in polarization at later inflammatory stages. Therefore, Notch signaling may stimulate the production of pro-oncogenic cytokines if the activated macrophages are immature, or M2 phenotype. Most TAMs often are poorly immunogenic or immunosuppressive [30]. Here we demonstrated that the chemokines produced by TAMs, e.g., CCL5, favored tumor invasion and glycolysis.

AMPK, a phylogenetically conserved serine/threonine protein kinase, is a central cellular energetic biosensor and regulator of a broad array of cellular metabolic routes. AMPK is activated by a decrease in the ATP/AMP ratio, which is a good indicator ofenergetic stress. Once activated, AMPK switches on ATP-producing catabolism, and switches off ATP-consuming anabolism [31]. For a long time AMPK was regarded as a tumor suppressor: the overall progress of cancers was closely associated with reduced AMPK activation [32], and inhibition of AMPK resulted in the increase of cancer cell proliferation [33]. AMPK activators, such as metformin and phenformin, were reported to delay tumor onset in tumor-prone mice [34]. As the activation of AMPK usually inhibits cellular protein biosynthetic pathways, which are essential for cancer cell proliferation, it is apparent that AMPK is a restriction at the onset of tumor. However, our results demonstrated that AMPK-mediated glycolysis played a key role in cancer cell EMT and migration. These data were in accordance with recent investigations, which suggested that AMPK might confer resistance to chemotherapy by activating autophagy [35], and facilitate cancer cell migration/invasion via regulation of EMT [36]. We postulated that activation of AMPK might confer metastatic advantages to cancer cells by sustaining cellular energetic viability, as evidenced by increased production of ATP.

Although we demonstrated a critical role of CCL5 in the metabolic communication between TAM-like macrophages and breast cancer cells, the role of other chemokines, e.g., CCL4, CXCL10 and CCL25, could not be totally ruled out as our results also showed that lactate increased the expression of these chemokines. CCL4 was also a ligand for CCR5. Despite the demonstration that CCL4 secretion was lower than that of CCL5, CCL4 was found to be produced by breast cancer cells (Supplementary Figure 26). Whether lactate enhanced the production of other cytokines, thereby promoting cancer glycolysis by regulation of host-tumor interactions, remains to be investigated.

It has long been known that tumor growth is promoted by TAMs. There is a strong correlation between macrophage density and poor patient prognosis [37]. Several cytokines, including CSF-1, GM-CSF, IL-3, and CCL2 [38], can mediate the biological function of TAMs, and therefore making them attractive targets for therapeutic purposes. We demonstrated that TAMs under the stimulation of lactate produced large sums of CCL5, which facilitated breast cancer glycolysis and EMT. CCL5 and its receptor CCR5 represent significant therapeutic targets that should be considered in the future treatment of breast cancer.

## MATERIALS AND METHODS

### Patients and specimens

Sections of paraffin-embedded tissue samples were provided by Jiangsu Cancer Hospital, Nanjing, China. They were obtained from patients with breast cancer. The age range was 33 to 74 years. All patients underwent curative-intent surgery at the Department of Surgery from 2010 to 2015. In total, 28 primary breast tumors and corresponding adjacent normal tissues were studied. Tumor stage was determined according to the 2009 International Union Against Cancer TNM Classification system (7th edition). None of the patients had received pre-operative chemotherapy or radiotherapy. Pathologists confirmed the histopathological diagnosis for each specimen. The study protocol was reviewed and approved by the ethics committee of China Pharmaceutical University.

### Chemicals and regents

Cell culture media (RPMI-1640, L-15 and MEM), fetal calf serum (FCS), and Lipofectamine 2000 were from Gibco/Invitrogen (Carlsbad, CA, USA). CCL5 (DRN00B), TGF-β1 (DB100B) ELISA kits and recombinant human CCL5 (278-RN/CF) and TGF-β1 (240-B) were bought from R&D Systems (Minneapolis, MN). The following antibodies were obtained from Cell Signaling Technology (Beverly, MA): CD68 (#76437, 1:200), AMPKα (#5831, 1:1200), p-AMPKα (Thr172) (#50081, 1:1000), NICD (#4147, 1:900), p-P65 (Ser536) (#3033, 1:1000), p-STAT3 (Tyr705) (#9145, 1:1000), HIF-1α (#79233, 1:800), E-cadherin (#3195, 1:1500), N-cadherin (#13116, 1:1000), Vimentin (#5741, 1:1500), PKM2 (#4053, 1:1200), LDHA (#3582, 1:1200), c-Myc (#5605, 1:1000), Akt (#4685, 1:1800), p-Akt (Ser473) (#4060, 1:1000), ACC (#3676, 1:1400), p-ACC (Ser79) (#11818, 1:1000), Jagged 1 (#70109, 1:1200), Jagged 2 (#2205, 1:1000), Notch 1 (#3608, 1:1000), Notch 2 (#5732, 1:1000). CCL5 (AF-278-NA, 15μg/ml), CCR5 (MAB1802, 1μg/ml; MAB181, 25μg/ml), TGF-β1 (MAB240, 20μg/ml) were purchased from R&D Systems. HK2 (sc-374091, 1:600) and β-actin (sc-8432, 1:1500) were commercially obtained from Santa Cruz (Santa Cruz, CA). Anti-CCL5 neutralizing antibody (500-M75, 5μg/ml) was from Peprotech (Rocky Hill, NJ). GSK 2837808A (#5189), DAPT (#2634), SIS3 (#5291), Compound C (#3093), Maraviroc (#3756) and 2-deoxy-D-glucose (2-DG) (#4515) were purchased from Tocris (Bristol, UK).

### Cell culture

Human breast cancer MDA-MB-231 cells was cultured as monolayers in L-15 medium supplemented with 10% fetal calf serum (FCS), 100 U/ml penicillin and 100 mg/ml streptomycin. Human breast carcinoma MCF-7 cells were maintained in Eagle's Minimum Essential Medium with 0.01 mg/ml insulin and 10% FCS. Human acute monocytic leukemia THP-1 cells were maintained in RPMI-1640 medium supplemented with 0.05 mM 2-mercaptoethanol and 10% FCS. All the cell lines were obtained from Shanghai Institute of Life Science, Chinese Academy of Sciences. THP-1 cells were differentiated into macrophages using a standard protocol [39, 40], by culturing the cells with 50 ng/ml of PMA for 48 h. In lactic acid stimulation experiments, the growth medium of control macrophages was titrated to pH6.1 using sterile HCl. Breast cancer cells were co-cultured with macrophages in 6 well cell culture insert plates and separated by transwell filters (0.4 μm). Briefly, 10^5^-10^6^ THP-1 macrophages were plated on the lower chamber and 10^5^-10^6^ breast cancer cells (Ratio 1:1) were seeded on the upper chamber, respectively.

### Monocyte isolation

The isolation of human Peripheral blood monocytes (PBMCs) was performed by Ficoll density gradient centrifugation as previously described [41, 42]. Isolated PBMCs were seeded at 2×10^6^/ml in 24-well plates in RPMI 1640 medium supplemented with 10% FCS and 2mM L-glutamine. After 7 days of culture, non-adherent cells were removed by repeated gentle washing with warm medium. More than 95% of the adherent cells obtained with this technique were CD14^+^ macrophages.

### Animal model

Female BALB/c athymic nude mice (5 weeks) with body weight from 18 to 22 g were purchased from the Model Animal Research Center of Nanjing University. Mice were maintained according to the guidelines for the welfare and use of animals in cancer research in a temperature-controlled room (22°C). THP-1 macrophages were firstly activated by 15 mM lactic acid, and then co-cultured with MDA-MB-231 cells for seven days. Culture media were changed every three days. After co-culture, breast cancer cells were collected and 3×10^5^ MDA-MB-231 cells were injected into the tail vein of nude mice. After two weeks, animals were sacrificed and metastatic nodules on lung surfaces were counted. To validate the effects of CCL5 in promoting lung metastasis, 5μg/ml anti-CCL5 neutralizing antibody was added to the co-culture system.

### Quantitative PCR

Total RNA from cells was extracted with TRIzol reagent (Invitrogen) and reverse transcription was performed using the cDNA synthesis system (Invitrogen). Quantitative polymerase chain reaction (PCR) was carried out on an iCycler Real-time PCR Detection System (Bio-Rad, Hercules, CA) as previously described [20]. The primers used for quantitative PCR were listed in Supplementary Table 3.

### Immunoblotting and immunofluorescence

Total cellular extracts were prepared using standard procedures. Western blotting and immunofluorescence were done as previously described [20]. The immunoblotting bands or dots were visualized with an enhanced chemiluminescence western blotting detection reagents (Amersham, UK). For immunofluorescence microscopy, the secondary antibody was fluorescein isothiocyanate (FITC)-conjugated goat anti-mouse IgG (Santa Cruz) and coverslips were examined on an inverted microscope (Zeiss Axiovert 200 M, Germany).

### ELISA

Cells were treated with different concentrations of lactic acid or cytokines, and the supernatants were harvested. Each supernatant was centrifuged at 2000 g and stored at -70°C until analysis. Enzyme-linked immunosorbent assay was performed according to the manufacturer's instructions (R&D Systems).

### Migration assay

Cell migration assays were conducted in a double chamber transwell assay. Briefly, 2-5×10^4^ breast cancer cells/well were seeded into the upper chamber, and chemoattractant was placed in the lower chamber. The positive control was 5% FCS; the negative control was base medium alone. After incubation at 37°C for 24 h, migrated cells were stained and counted in five randomly selected fields.

### Glucose uptake, lactate production and ATP levels

The levels of lactic acid in the culture medium were measured using the L-Lactate Colorimetric Assay Kit (Abcam). Briefly, breast cancer cells were co-cultured with human macrophages for 72 h, and then transferred to a 90-mm dish. Fresh media were added and cells were further cultured for 12-24 h. After that, the lactic acid levels were measured according to the manufacturer's instructions.

Glucose uptake was measured using the Amplex Red Assay. Cells were treated as described in lactate assay and the amount of glucose was detected according to the manufacturer's instructions. Glucose uptake was determined by subtracting the amount of glucose in each sample from the total amount of glucose in the fresh medium.

ATP levels were determined using the Kinase-Glo® Max Luminescent Kinase Assay kit (Promega) as we described in our previous work [43].

### Luciferase reporter gene assay

The construction of pGL3-CCR5 vector (containing 1040bp CCR5 core promoter), and cell transfection were described in our previous work [20]. To investigate the mechanism of CCR5 gene transcription, cells were co-transfected with pGL3-CCR5 and renilla luciferase reporter vector pRL-TK. Cells were then treated with different concentrations of TGF-β1 for 24 h, and luciferase activities were determined.

### Plasmids construction and cell transfection

The construction of pcDNA3.1-CCR5 vector (containing the full length of human CCR5 gene) was described in our previous work [20]. To select stable transfectants, 48 h after transfection, cells were passaged and grown in medium containing G418.

### Short hairpin RNA and RNA interference

The construction of CCR5 siRNA vectors was reported in our previous work [20]. The shRNA sequences designed against human CCR5 were as follows (targeting sequence): shCCR5-1, 5’-GAGCATGACTGACATCTAC-3’, shCC R5-2, 5’-CTCTGCTTCGGTGTCGAAA-3’. The validated siRNAs were: Notch1, 5’- AAGGUGUCUUCCAGAUC CUGA -3’ [44]; ALK5, 5′-CAUAUUGCUGCAACCA GGATT-3′ [45] and AMPKα1, 5′-UGACAAGCACUUA CUCCAATT-3′ [46]. Gene knockdown was confirmed by both quantitative PCR and western blot.

### Immunohistochemical analysis

Immunohistochemical studies were performed in a standard protocol as described in our previous work [20].

### Statistical analysis

Results were mean ± SEM of three independent experiments. Unpaired Student's t-test and Spearman correlation coefficient analysis were used. P values were two-sided: 0.05 was considered statistically significant.

## SUPPLEMENTARY MATERIALS FIGURES AND TABLES




