# Supplementary material for: Lactate-activated macrophages induced aerobic glycolysis and epithelial-mesenchymal transition in breast cancer by regulation of CCL5-CCR5 axis: a positive metabolic feedback loop
Source: Oncotarget. 2017 Nov 30;8(66):110426–43. doi: 10.18632/oncotarget.22786 (PMC5746394; doi:10.18632/oncotarget.22786)
Supplement: Supplementary file 1 [file oncotarget-08-110426-s001.pdf]

Lactate-activated macrophages induced aerobic glycolysis and epithelial-mesenchymal transition in breast cancer by regulation of CCL5-CCR5 axis: a positive metabolic feedback loop

SUPPLEMENTARY MATERIALS

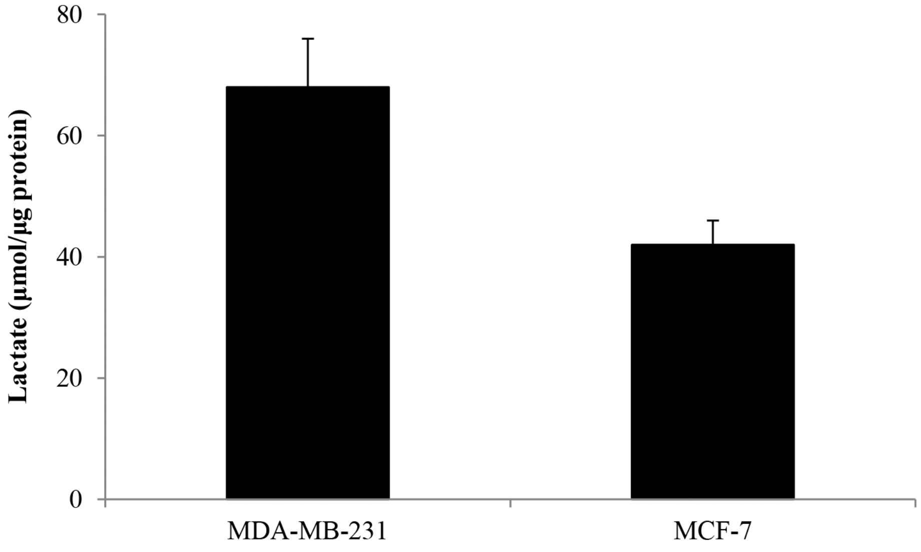

Supplementary Figure 1: The secretion of lactic acid in breast cancer cells. 10^6 cells were placed in 90-mm dishes, and the medium was changed after 24 h. Cells were further cultured for 24 h, and supernatants were collected to measure the levels of lactic acid.

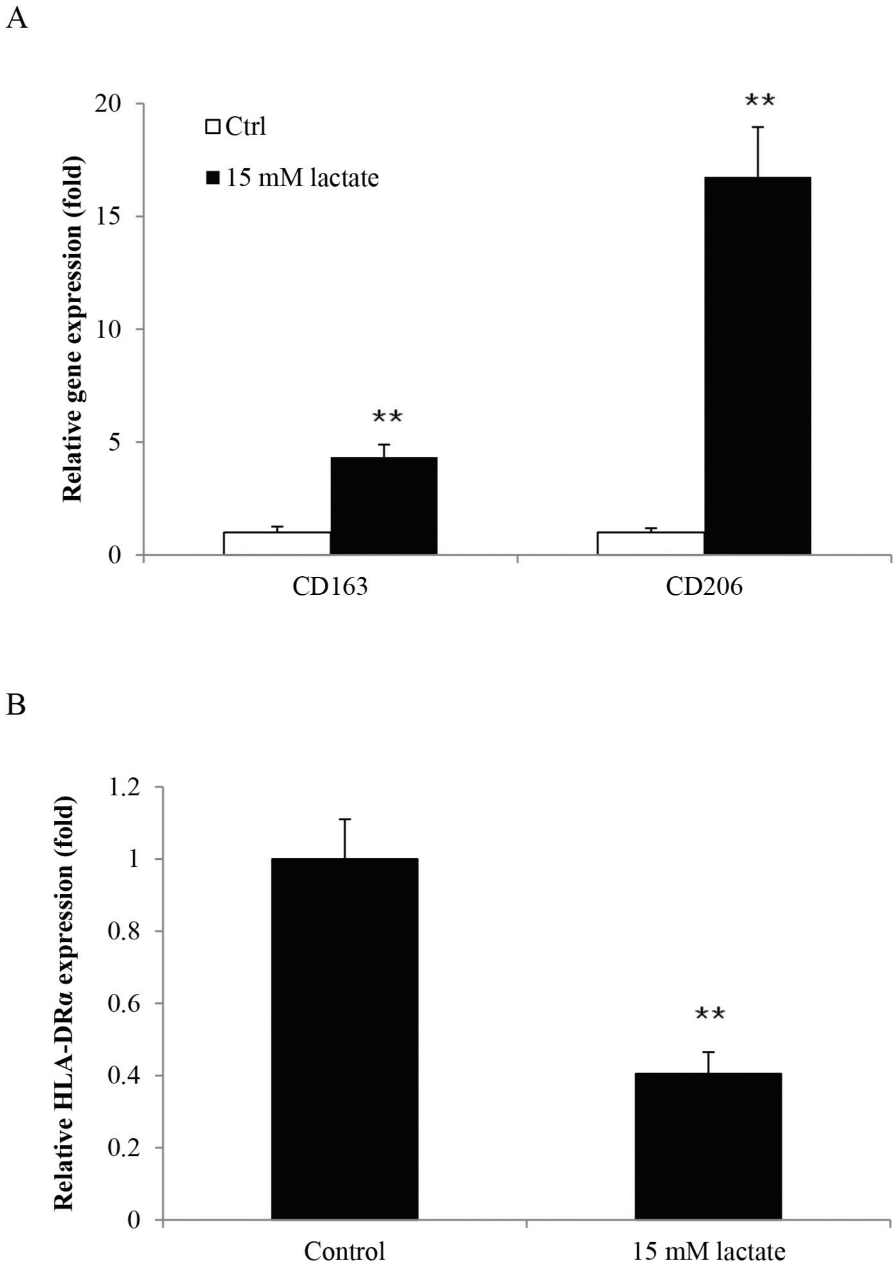

Supplementary Figure 2: Lactic acid polarized THP-1 macrophages to a M2 phenotype. **(A)** 10^6 THP-1 macrophages were treated with 15mM lactate for 72 h, and M2 macrophage marker CD163 and CD206 were investigated by quantitative PCR. **(B)** M1 macrophage marker HLA-DRα was decreased after 72 h lactate treatment.

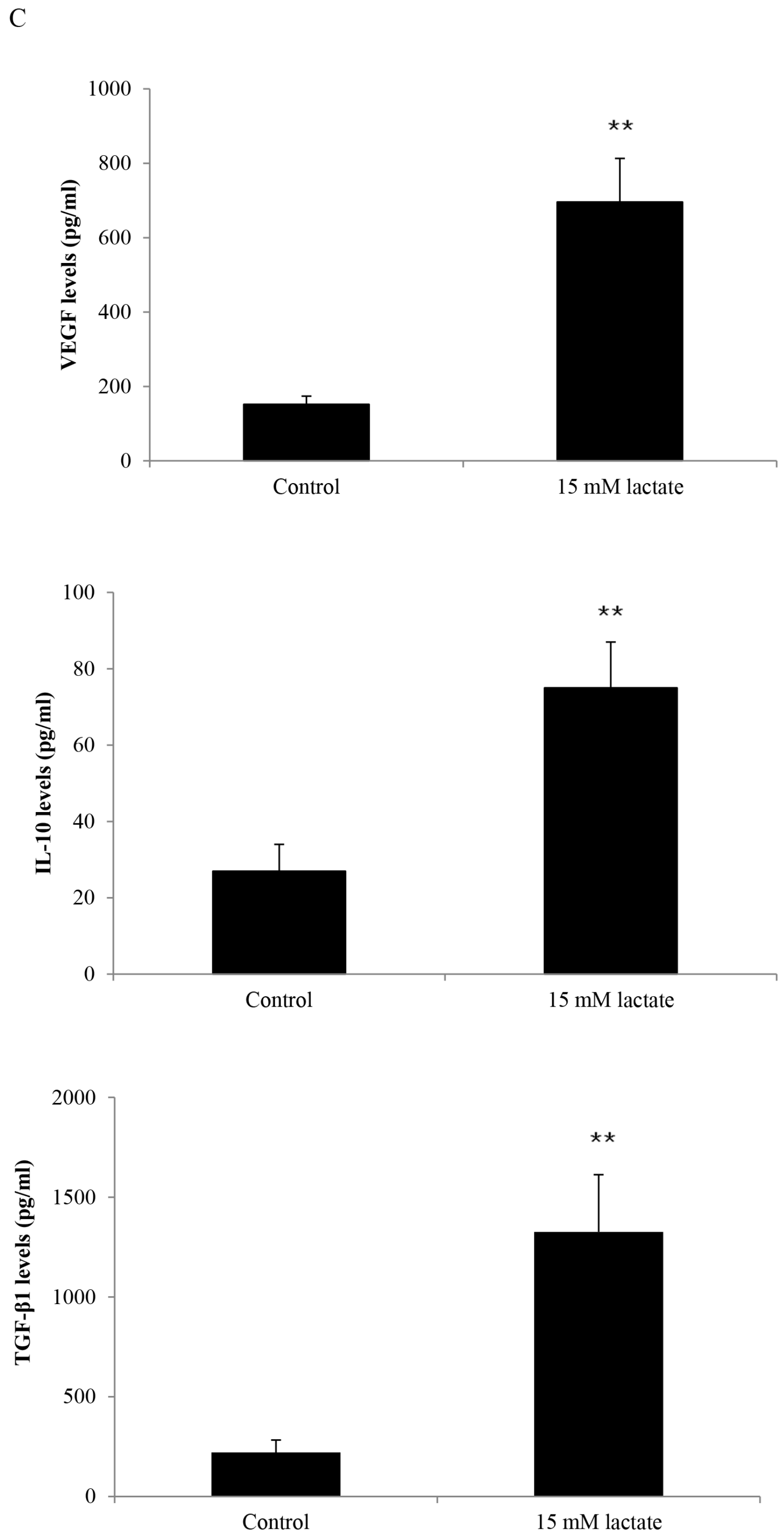

Supplementary Figure 2 (*Continued*): (C) TGF-β1, IL-10 and VEGF secretion were detected by ELISA assay after 72 h lactate treatment.

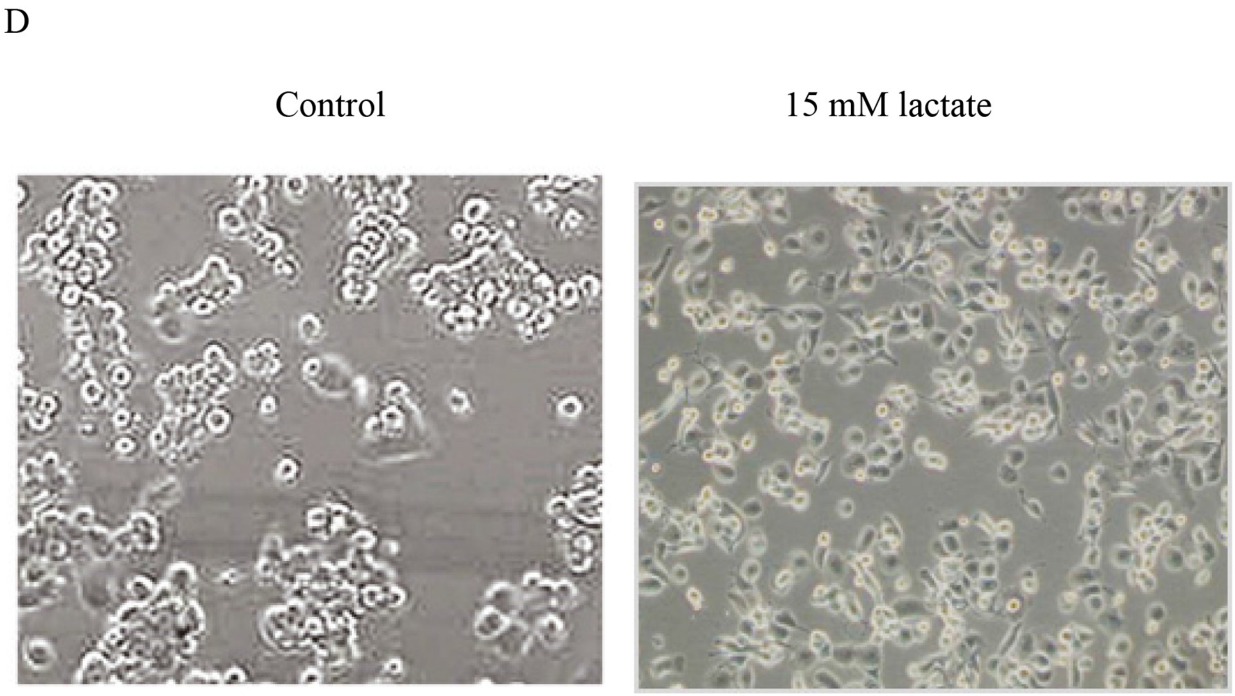

Supplementary Figure 2 (*Continued*): (D) THP-1 macrophages were stimulated with 15mM lactate for 72 h, and morphological changes were shown.

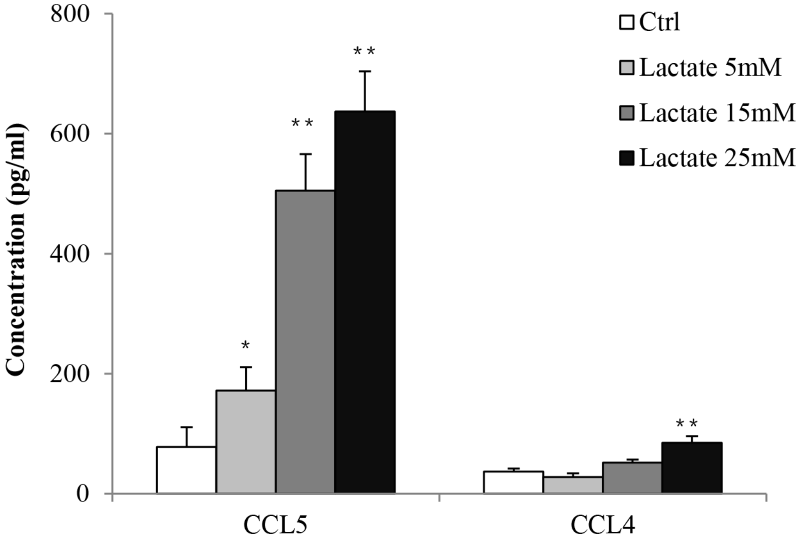

Supplementary Figure 3: The effect of lactic acid on the production of CCL4 and CCL5 in THP-1 macrophages. 10^6 THP-1 macrophages were treated with increasing concentrations of lactate for 48 h, and the levels of CCL4 and CCL5 were measured by ELISA. Control cells were cultured with the pH6.1 growth medium.

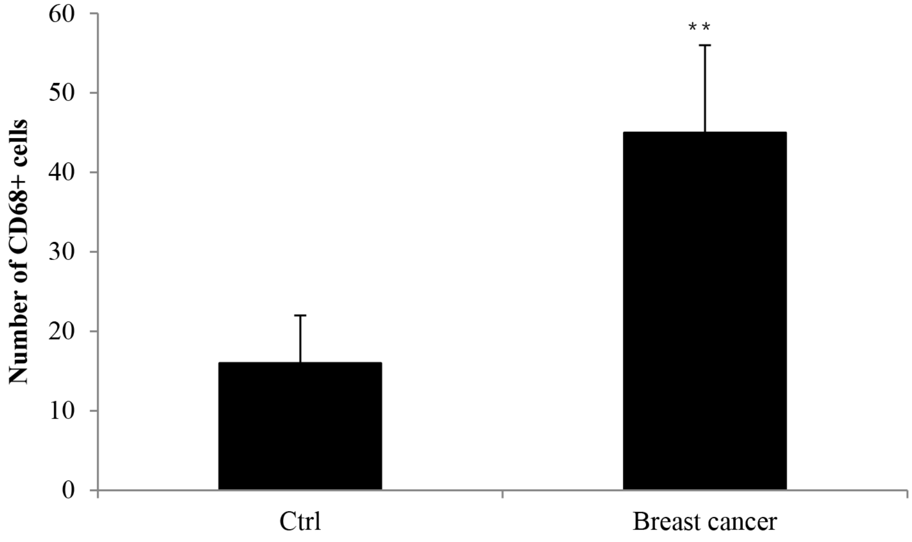

Supplementary Figure 4: The counting of $CD68^+$ macrophages in tumor adjacent tissues (control) and breast tumors (n=28).

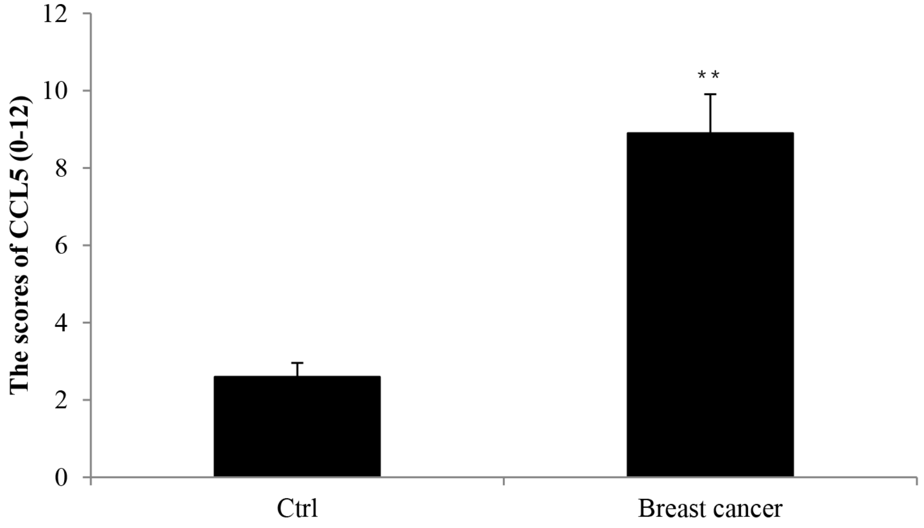

Supplementary Figure 5: Immunohistochemistry scores of CCL5 in tumor adjacent tissues (control) and breast tumors (n=28).

A

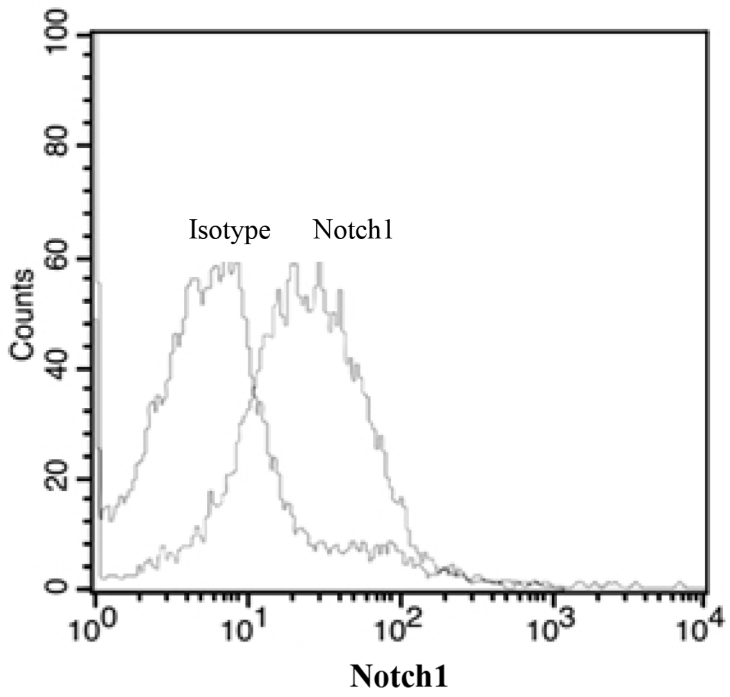

B

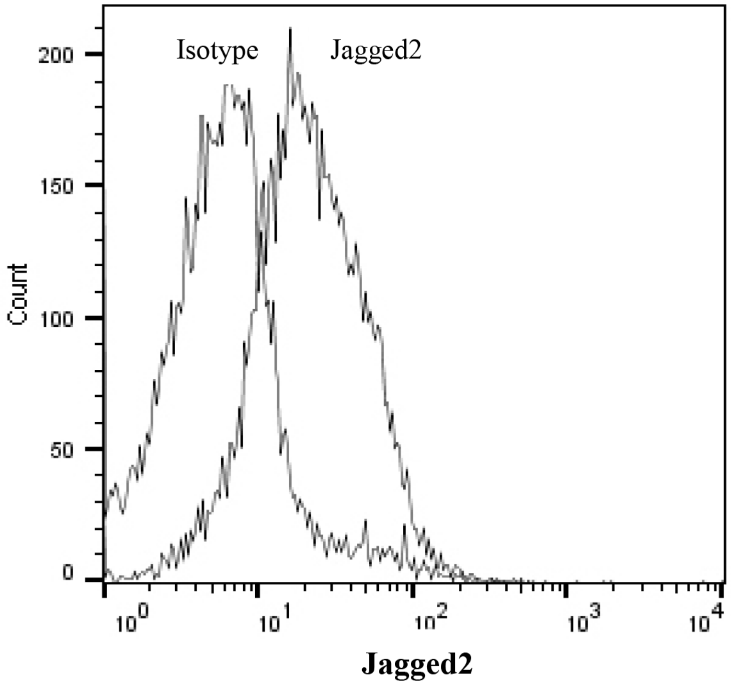

Supplementary Figure 6: The membrane expression of notch1 **(A)** and jagged2 **(B)** in THP-1 macrophages. Cells were collected and analyzed by flow cytometry.

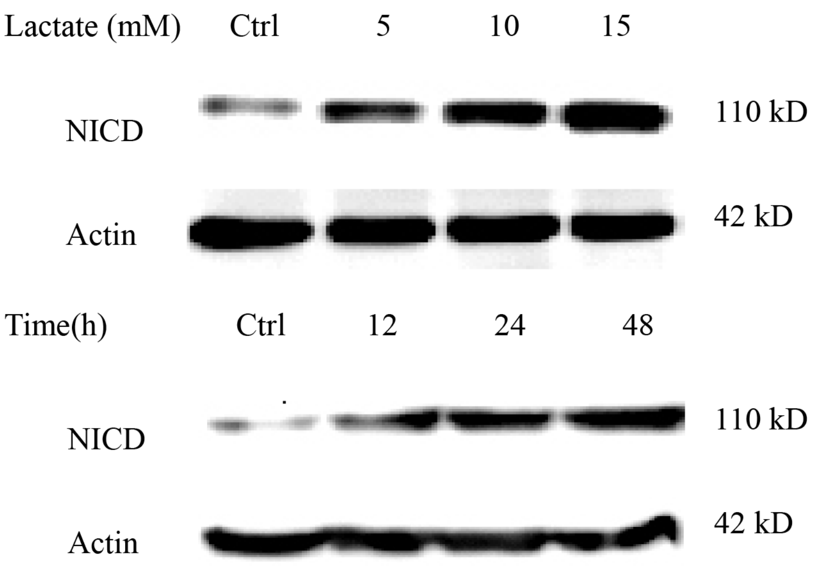

Supplementary Figure 7: Lactate increased the expression of NICD in primary macrophages. 10^6 primary macrophages were treated with increasing concentrations of lactate, or administrated with 15 mM lactate for different time points, and the protein levels of NICD were detected by western blot.

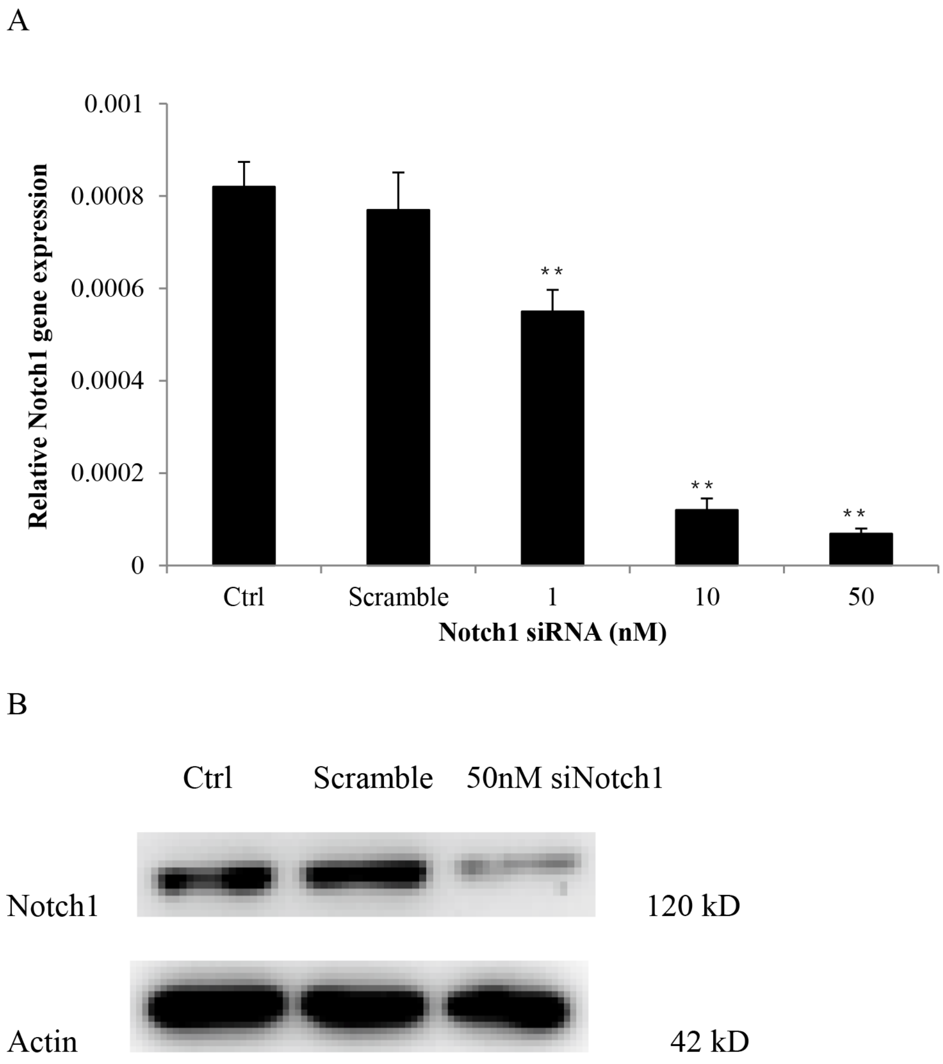

Supplementary Figure 8: Inhibition of Notch1 by double-strand siRNA specifically designed against human Notch1. **(A)** 3×10^5 THP-1 cells were transiently transfected with different concentrations of Notch siRNA for 24 h, and the gene expression of Notch1 was measured by quantitative PCR. **(B)** The protein levels of Notch1 were shown in THP-1 cells (10^6) after transfected with 50nM siNotch1.

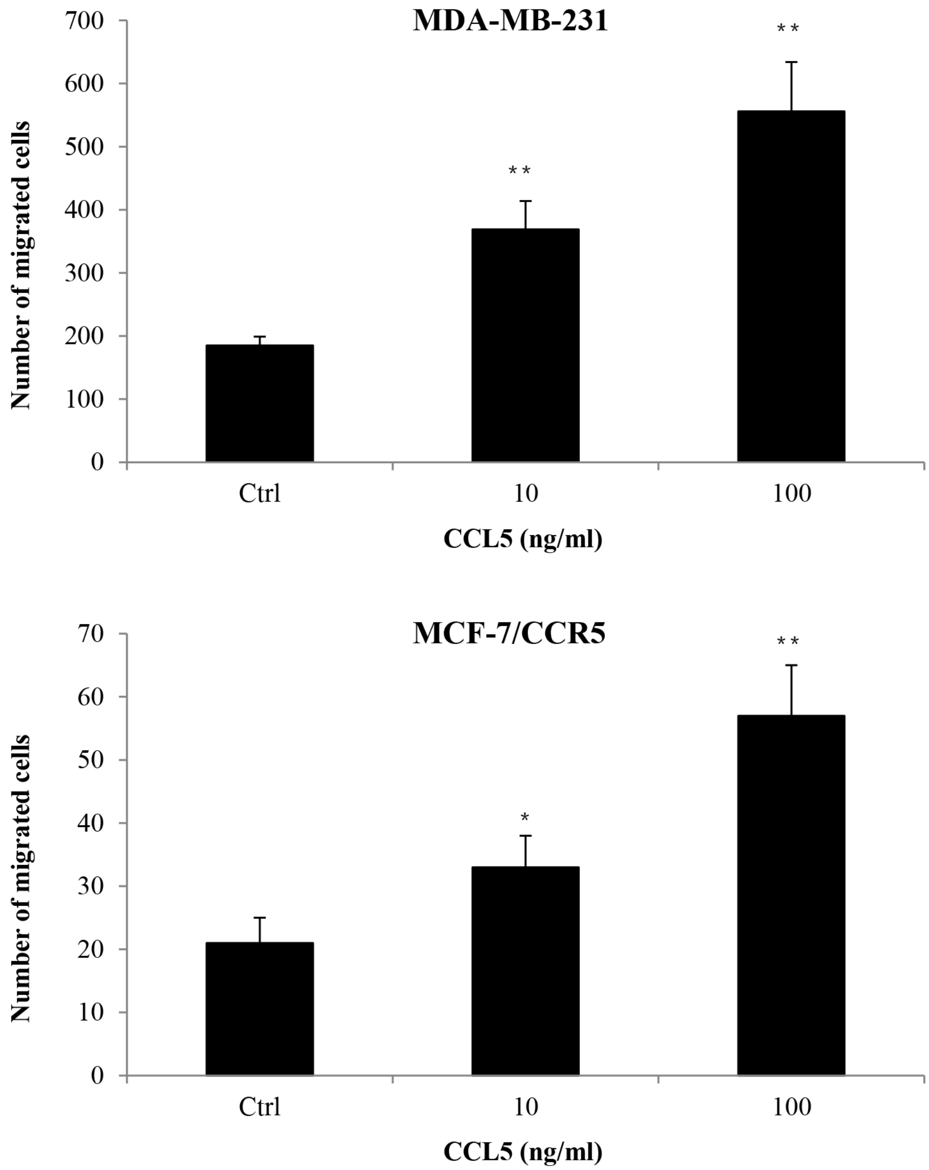

Supplementary Figure 9: CCL5 increased breast cancer cell migration (Transwell). 2×10^4 MDA-MB-231 or 5×10^4 MCF-7/CCR5 cells were placed in the upper chamber, and different concentrations of CCL5 were loaded in the lower chamber. After 24 hours, migrated cells were stained and counted.

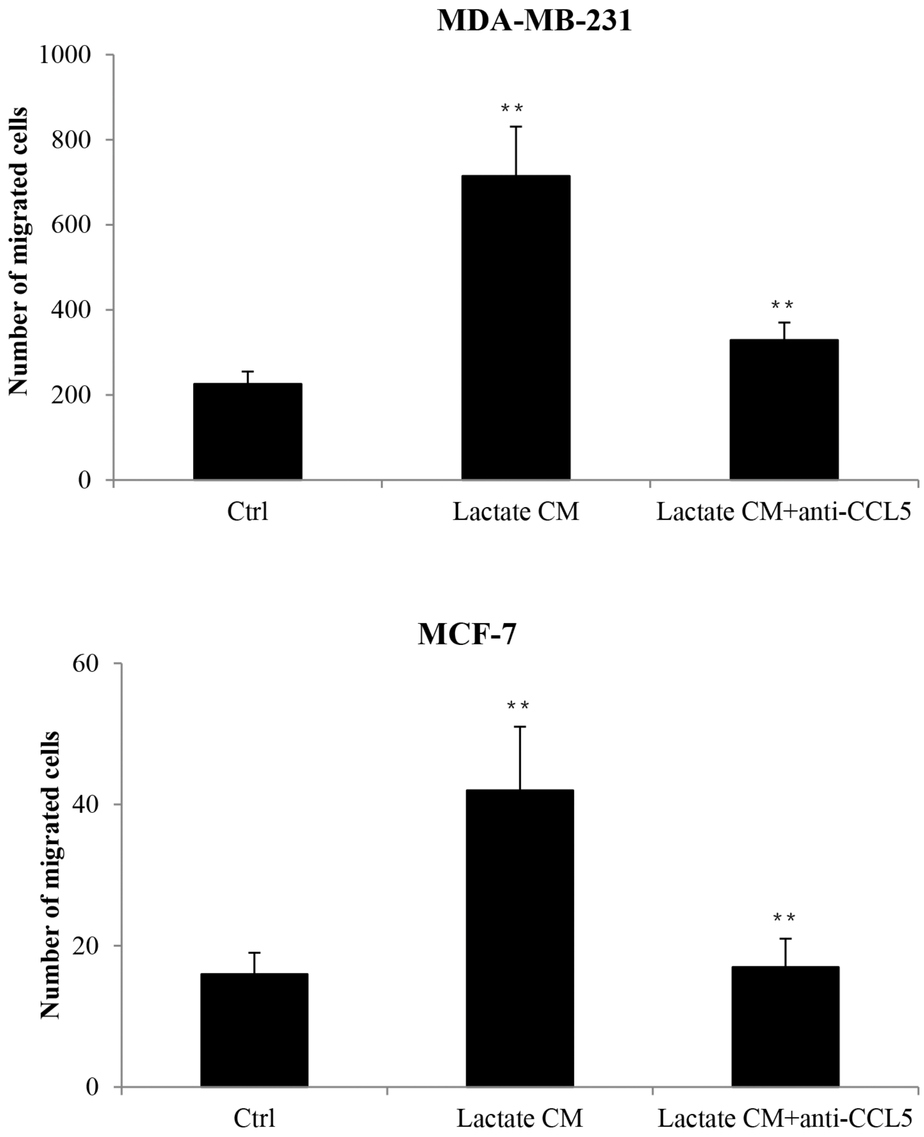

Supplementary Figure 10: Neutralization of CCL5 decreased macrophage-induced breast cancer cell migration. 10^6 THP-1 macrophages were treated with 15 mM lactate for 72 h, and then cells were washed twice and fresh media were added. Macrophages were cultured for another 24 h and the conditional media (CM) was collected. The effect of CM on breast cancer cell migration was measured by double chamber transwell assay. Briefly, 2×10^4 MDA-MB-231 or 5×10^4 MCF-7 cells were placed in the upper chamber, and the lower chamber was lactate CM. 5μg/ml anti-CCL5 neutralizing antibody was used.

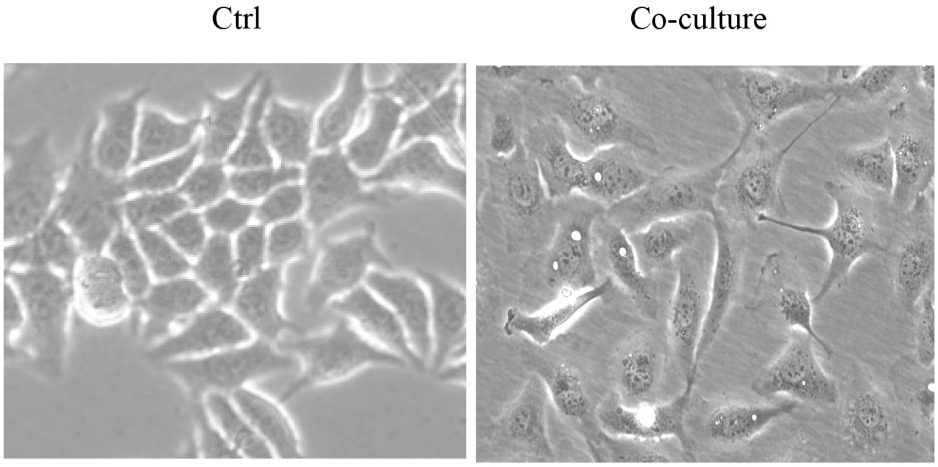

Supplementary Figure 11: Lactate-activated macrophages induced EMT in luminal MCF-7 cells. MCF-7 cells were co-cultured with 15 mM lactate-activated macrophages for 72 h, and cell morphological changes were shown.

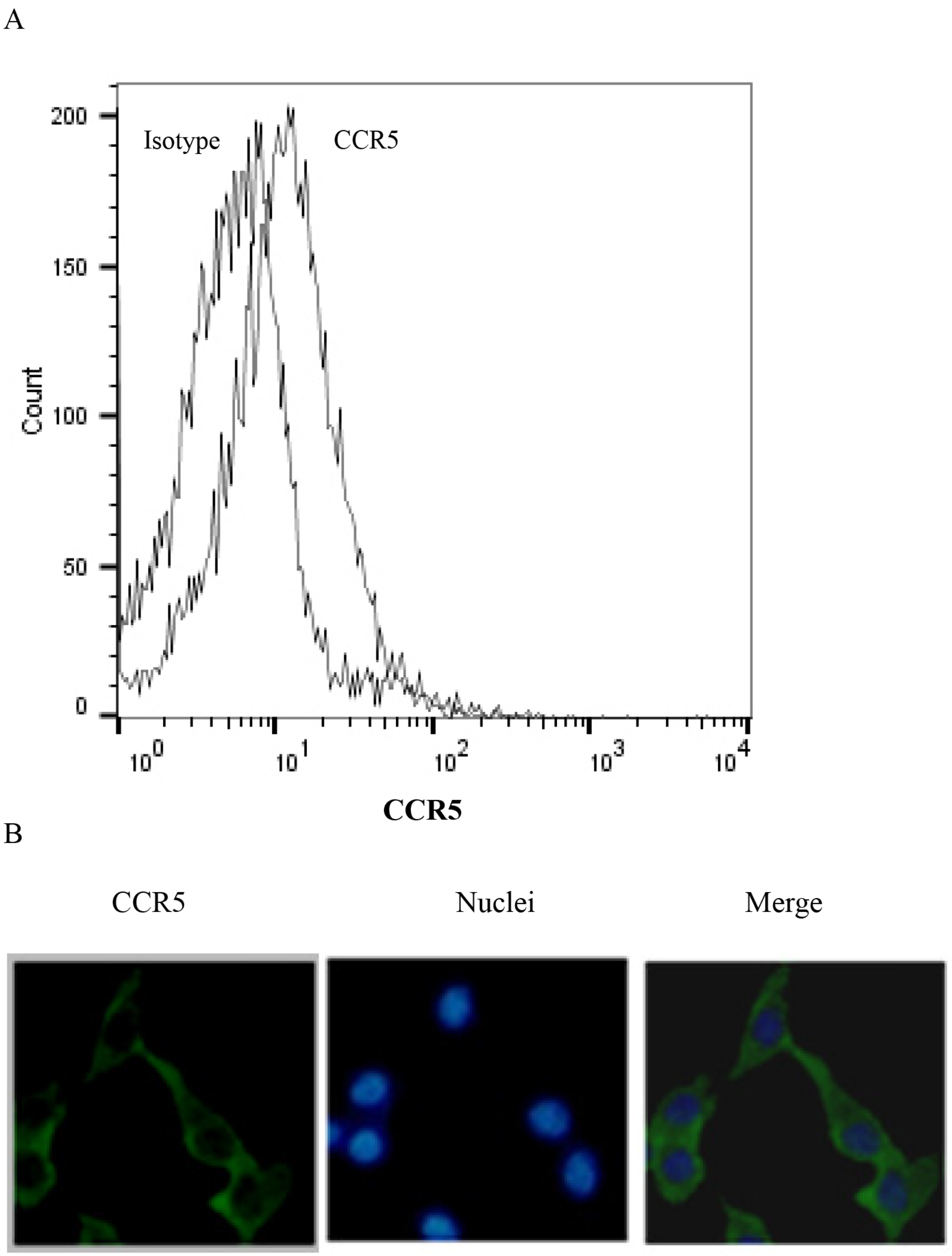

Supplementary Figure 12: CCR5 expression in MDA-MB-231 cells. The expression of CCR5 was detected by flow cytometry **(A)** and immunofluorescence staining **(B)**.

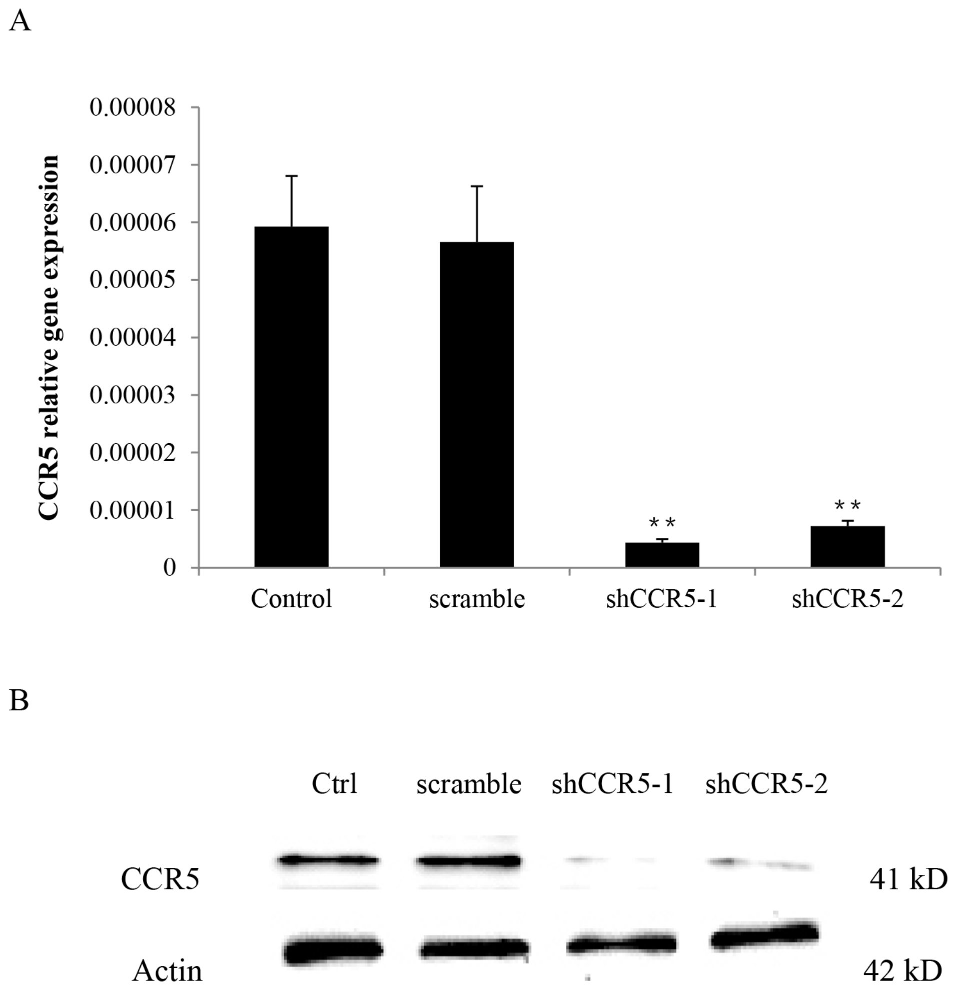

Supplementary Figure 13: Knockdown of CCR5 by short hairpin RNA specifically designed against human CCR5. (A) Expression of levels of CCR5 mRNA in MDA-MB-231 cells transfected with two kinds of shCCR5s (shCCR5-1 and shCCR5-2). Relative gene expression was measured by quantitative PCR. **(B)** CCR5 protein was detected by Western blotting in MDA-MB-231 cells transfected with shCCR5-1 and shCCR5-2.

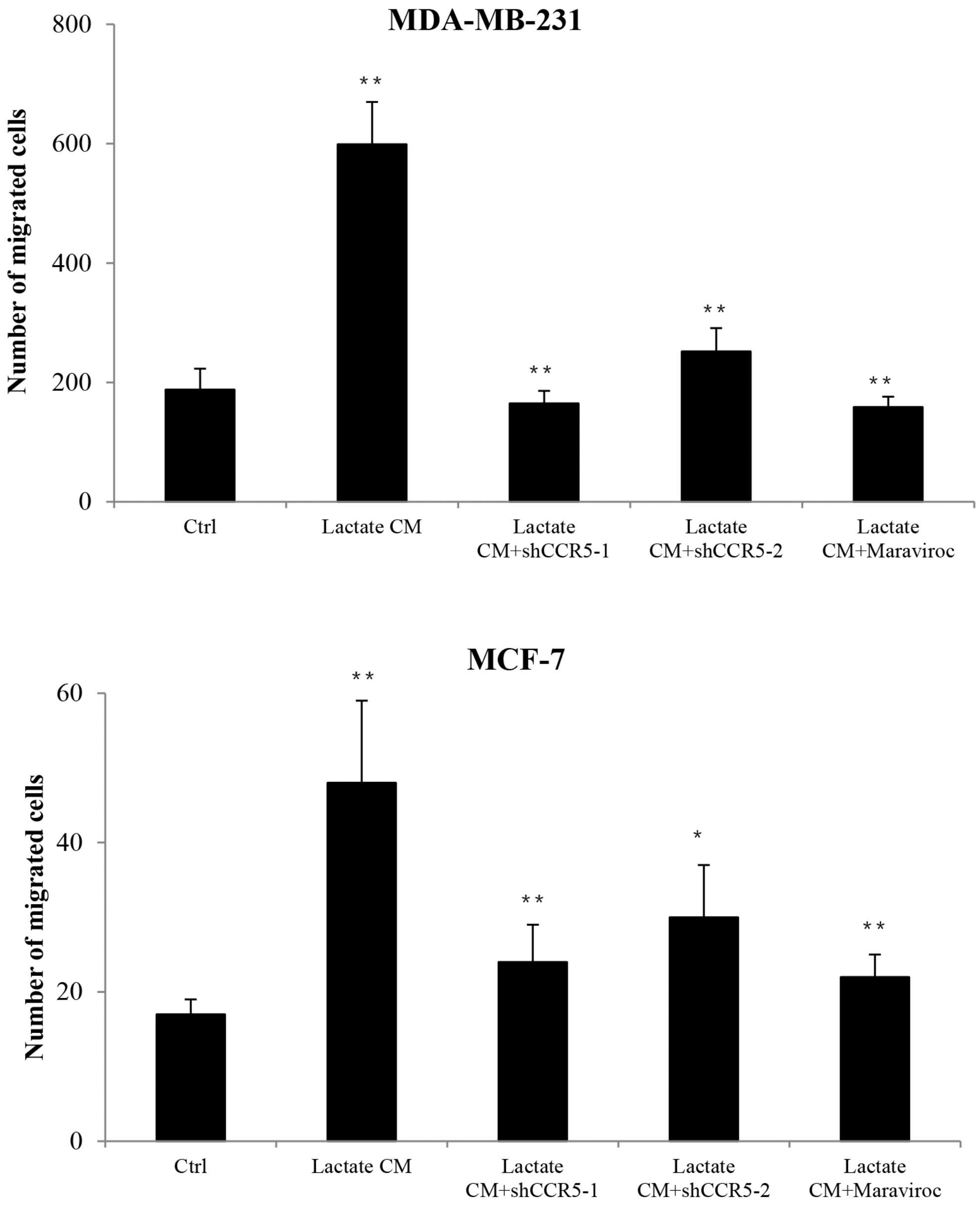

Supplementary Figure 14: Inhibition of CCR5 significantly reduced macrophage-induced breast cancer cell migration. Breast cancer cells were transfected with shRNAs designed against CCR5, or pre-treated with 5 μM Maraviroc for 2 h, then plated in the upper chamber of Transwll. The chemoattractant was latate CM collected from lactate-activated macrophages. After 24 hours, migrated cells were counted.

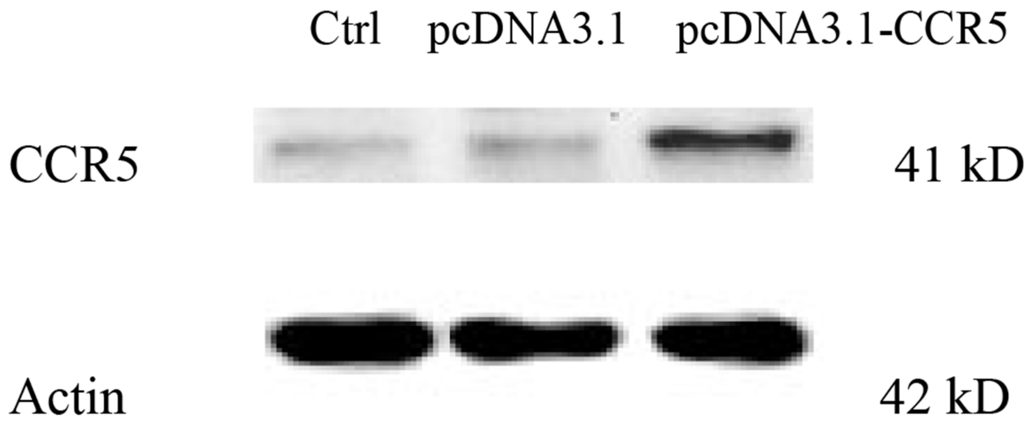

Supplementary Figure 15: Western blotting for CCR5 protein in MCF-7 cells transfected with pcDNA3.1-CCR5. Results presented were representatives of at least three independent experiments.

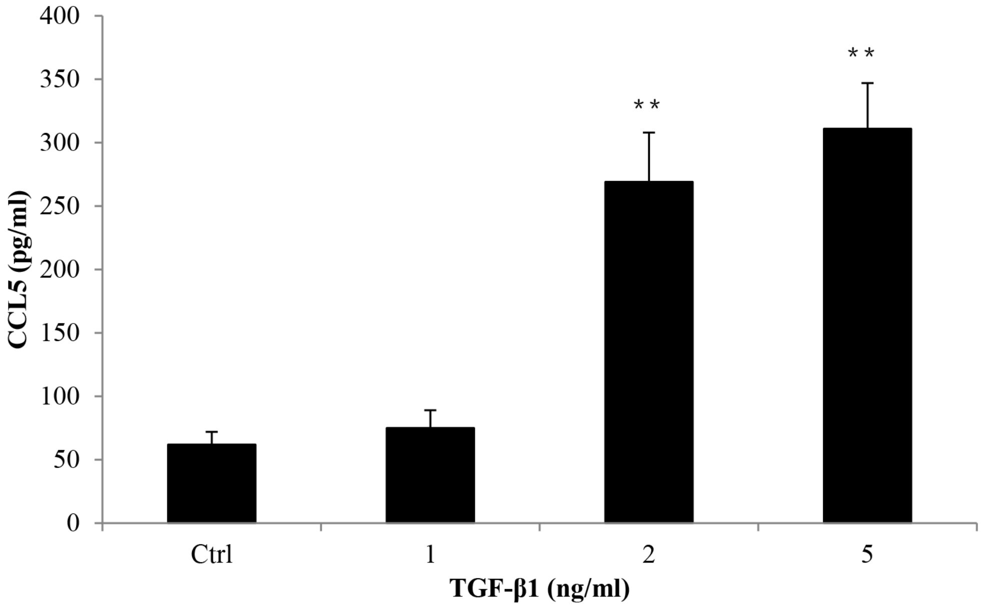

Supplementary Figure 16: TGF-β1 increased the production of CCL5 in macrophages. 10^6 THP-1 macrophages were treated with different concentrations of TGF-β1 for 48 h, and the production of CCL5 was measured by ELISA.

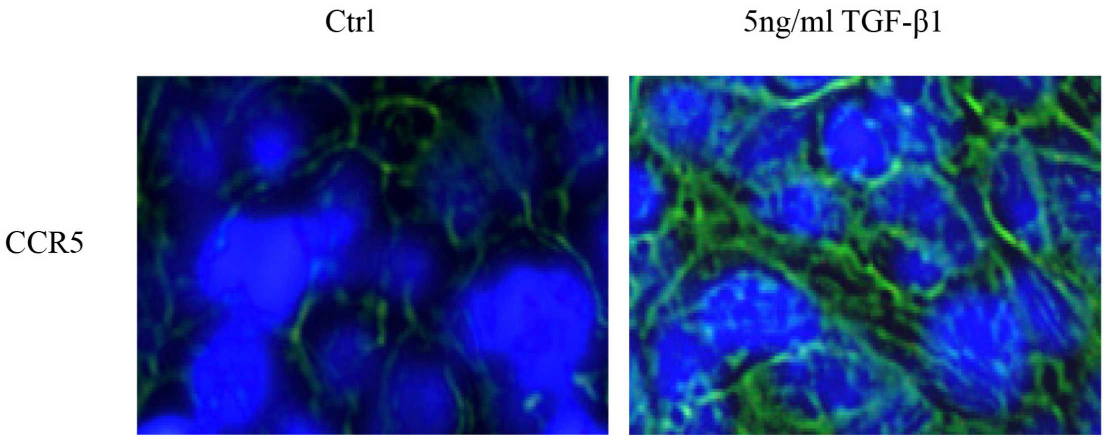

Supplementary Figure 17: TGF-β increased the expression of CCR5 in MCF-7 cells. Cells were stimulated with 5ng/ml TGF-β1 for 48 h, and immunofluorescence staining of CCR5 assayed.

A

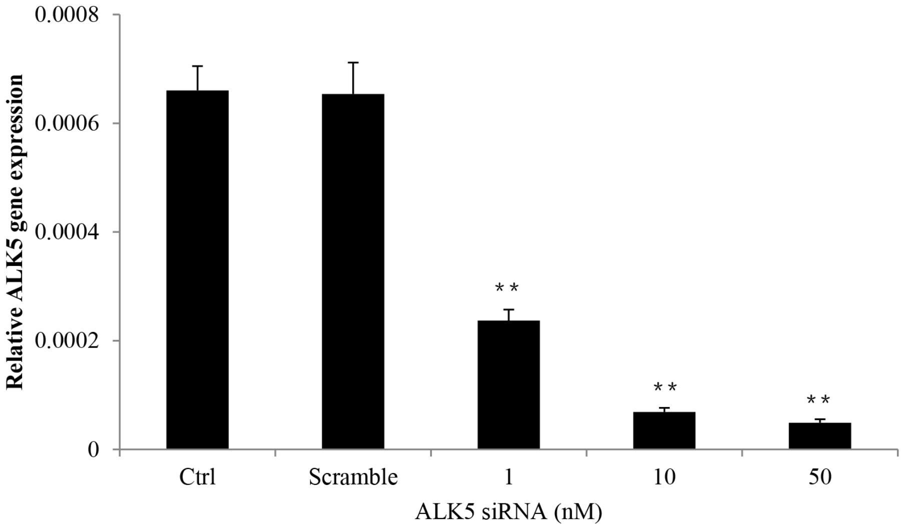

B

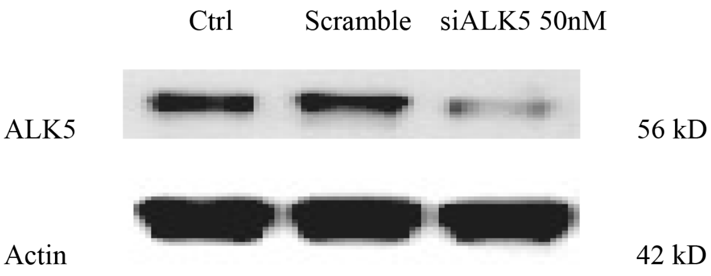

Supplementary Figure 18: Knockdown of ALK5 by double-strand siRNA specifically designed against human ALK5. **(A)** Expression of ALK5 mRNA in MCF-7 cells transfected with different concentrations of ALK5 siRNA. Relative gene expression was measured by quantitative PCR. **(B)** ALK5 protein was detected by Western blotting in MCF-7 cells transfected with 50 nM ALK5 siRNA.

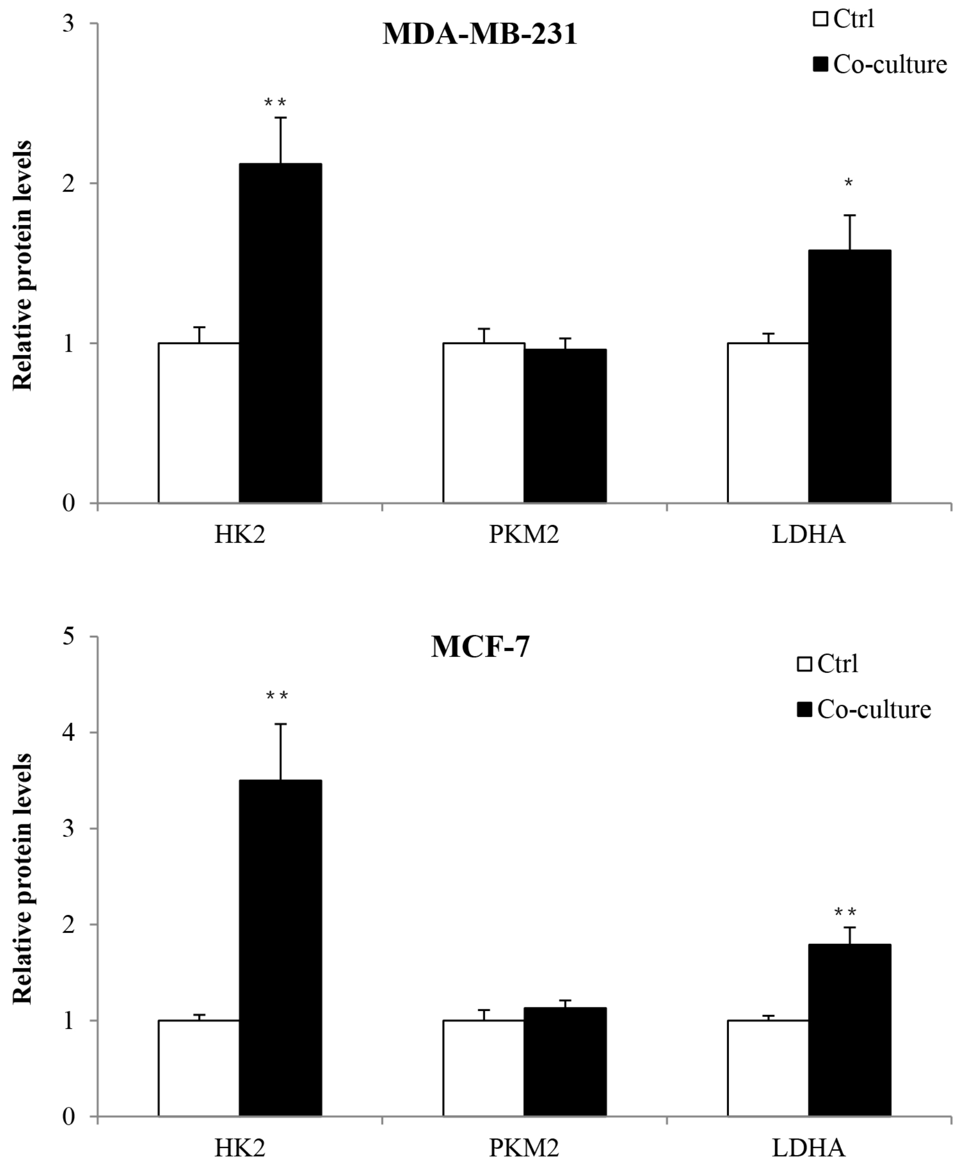

Supplementary Figure 19: Macrophages increased the expression of glycolytic proteins in breast cancer cells. Cells were co-cultured with 15 mM lactate-activated macrophages for 72 h, and then the protein expression of glycolytic enzymes was measured by western blot. The relative protein expression was quantified by densitomitric analysis.

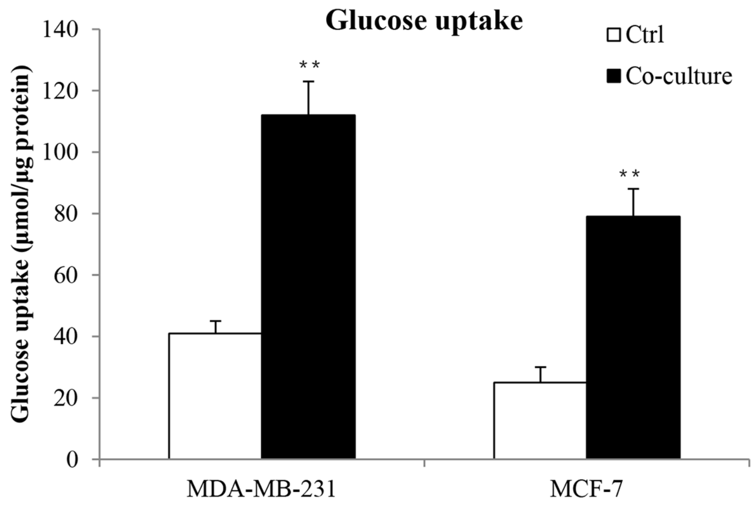

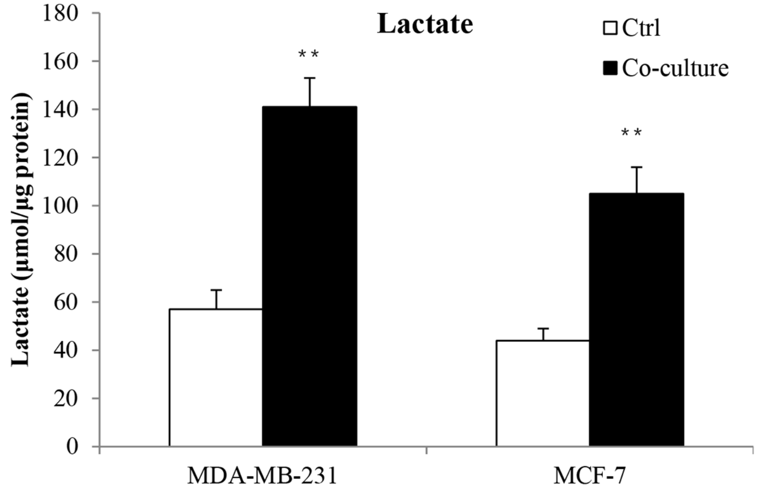

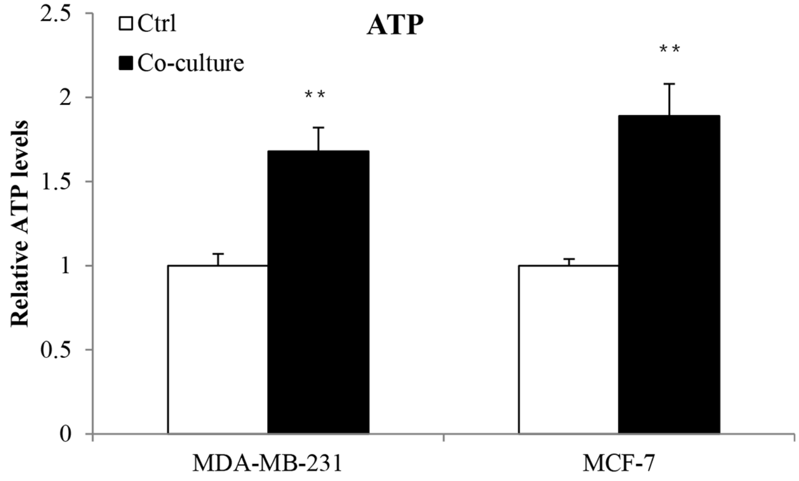

Supplementary Figure 20: Primary macrophages induced glycolysis in breast cancer cells. Primary macrophages were activated by 15 mM lactic acid, and then co-cultured with breast cancer cells for 72 h. After co-culture, breast cancer cells were washed and fresh media were added to culture for another 24 h. Glucose uptake, lactate production and ATP levels were then detected.

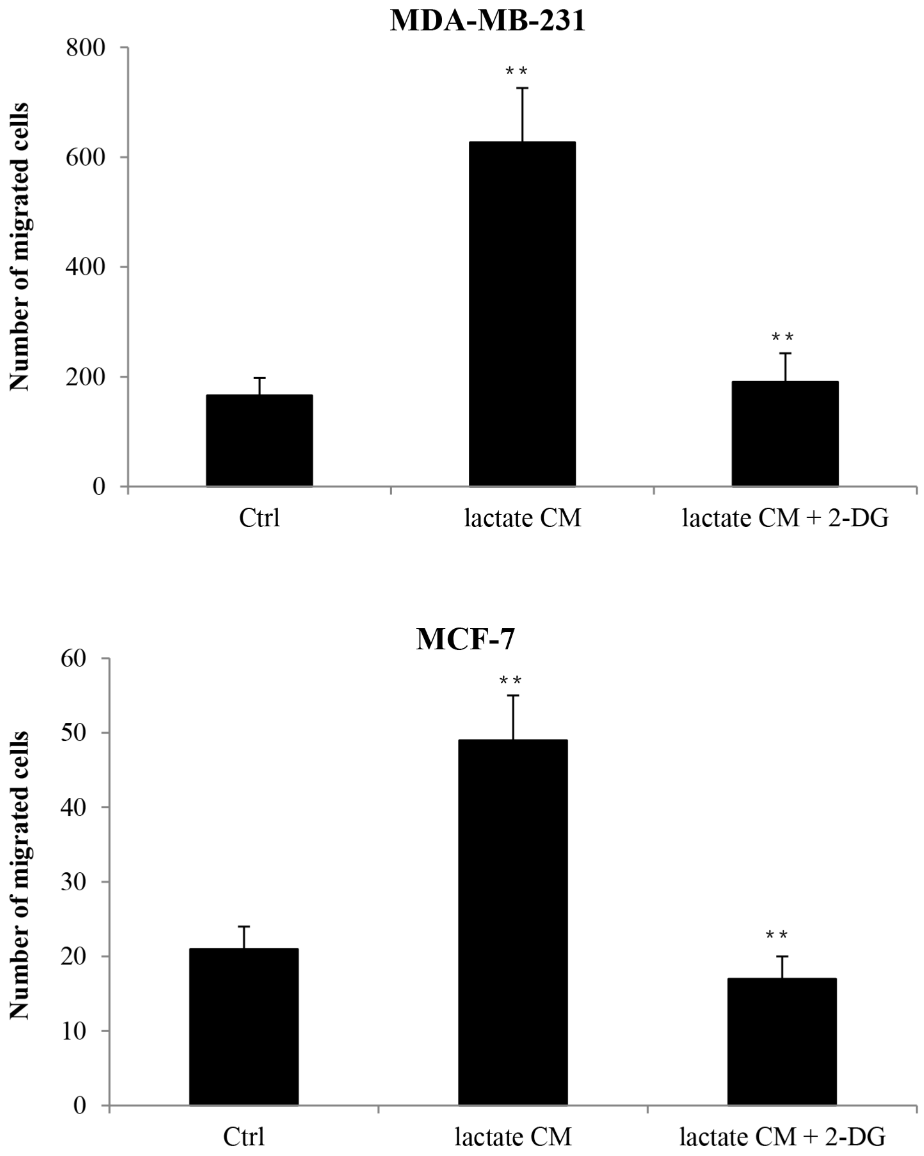

Supplementary Figure 21: Inhibition of glycolysis decreased macrophage-induced cell migration. 10^6 THP-1 macrophages were treated with 15 mM lactate for 72 h, and then cells were washed twice and fresh media were added. Macrophages were cultured for another 24 h and the conditional media (lactate CM) was collected. Breast cancer cells were pre-treated with 10 mM 2-DG for 4 h; then cell migration induced by lactate CM was detected by double chamber transwell assay.

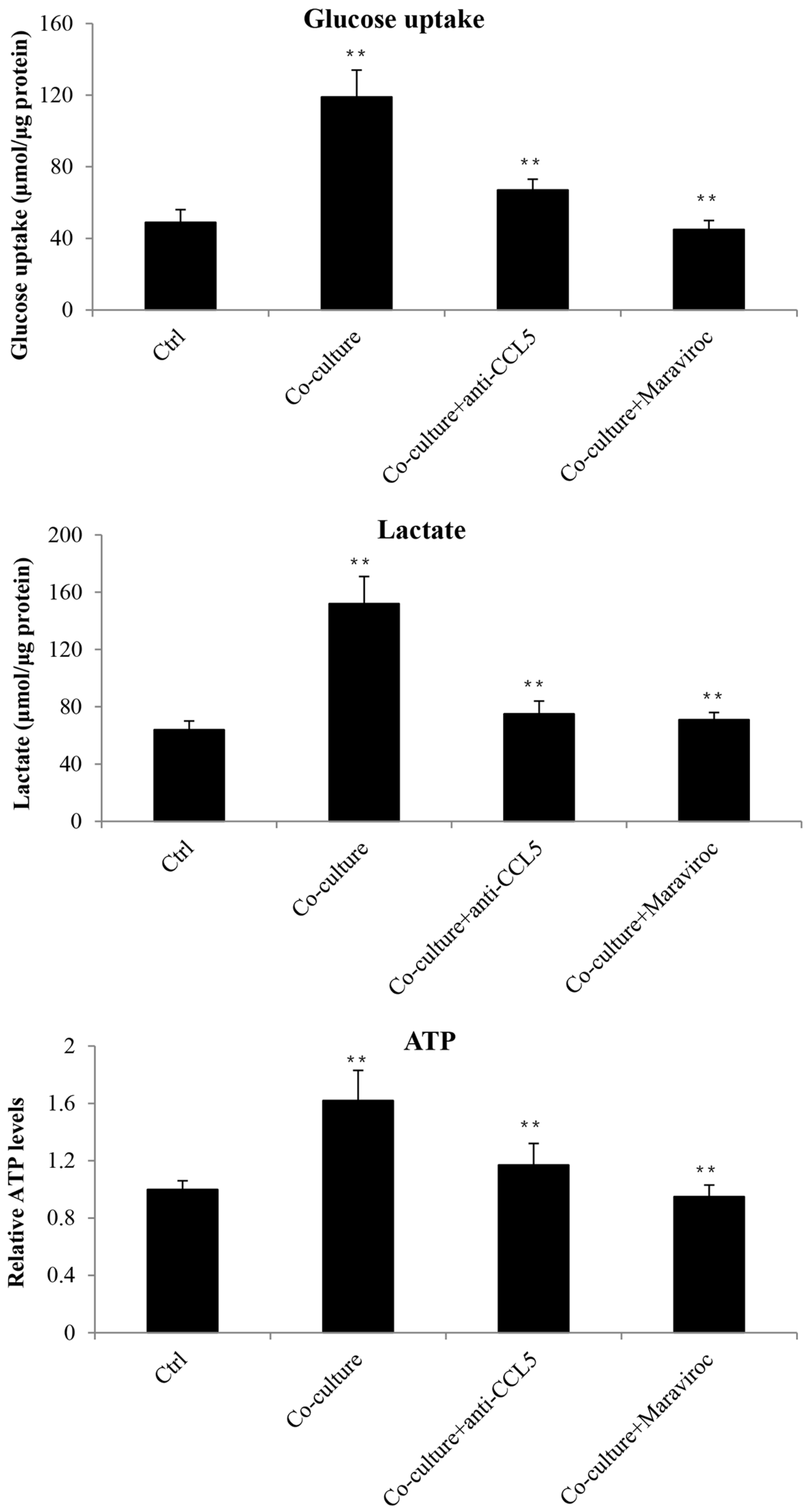

Supplementary Figure 22: Inhibition of CCL5-CCR5 axis reduced primary macrophage-induced glycolysis. MDA-MB-231 cells were pre-treated with 5μg/ml anti-CCL5 neutralizing antibody, or 5μM Maraviroc for 2 h, and then co-cultured with 15 mM lactate activated primary macrophages for 72 h. After co-culture, the media of MDA-MB-231 cells were changed and cells were further cultured for 24 h. Glucose uptake, lactate production and ATP levels were then investigated after 24 h.

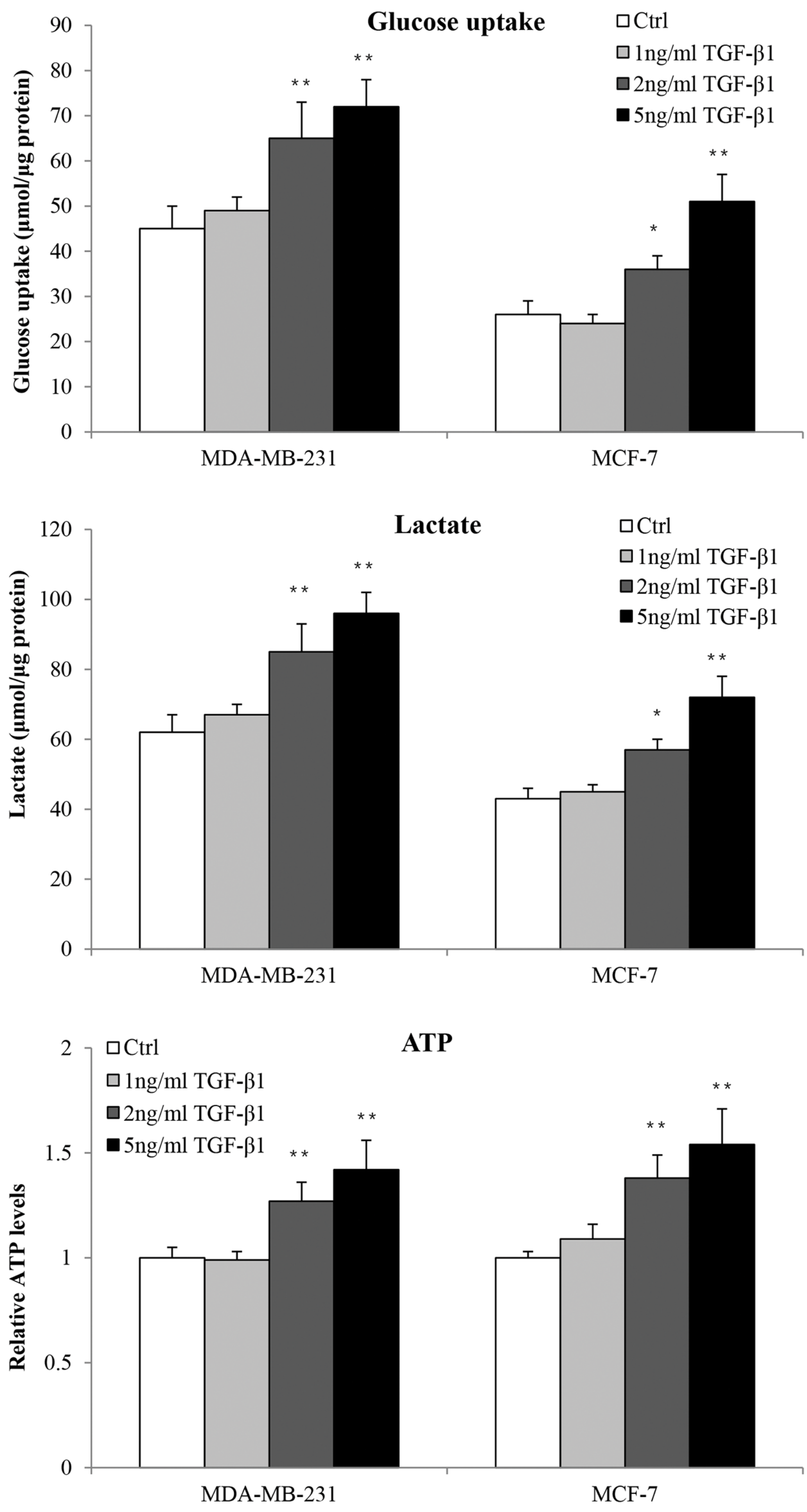

Supplementary Figure 23: TGF-β1 induced glycolysis in breast cancer cells. 10^6 Cells were treated with increasing concentrations of TGF-β1 for 12 h, and glucose uptake, lactate secretion and ATP levels were measured.

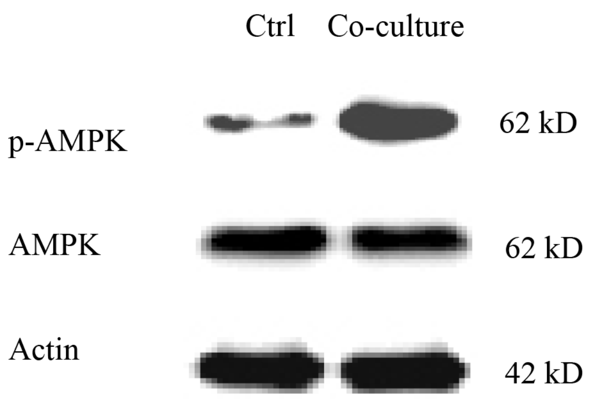

Supplementary Figure 24: Primary macrophages increased the phosphorylation of AMPK in MDA-MB-231 cells. Primary macrophages were activated, and then co-cultured with MDA-MB-231 cells for 72 h. The phosphorylation of AMPK in MDA-MB-231 cells was measured by western blot.

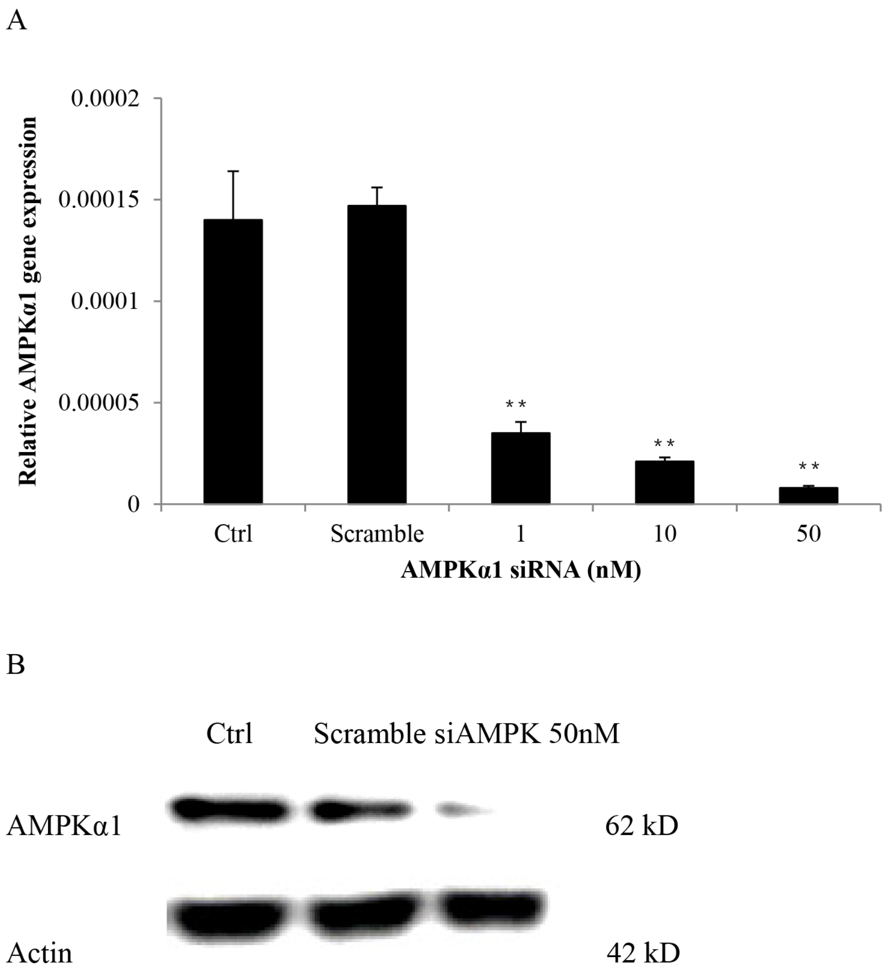

Supplementary Figure 25: Silencing of AMPKα1 in MDA-MB-231 cells. **(A)** 3×10^5 MDA-MB-231 cells were transfected with different concentrations of AMPKα1 siRNA, and AMPKα1 mRNA was measured by quantitative PCR. **(B)** AMPKα1 protein was detected by western blotting in MDA-MB-231 cells (10^6) transfected with 50 nM AMPKα1 siRNA.

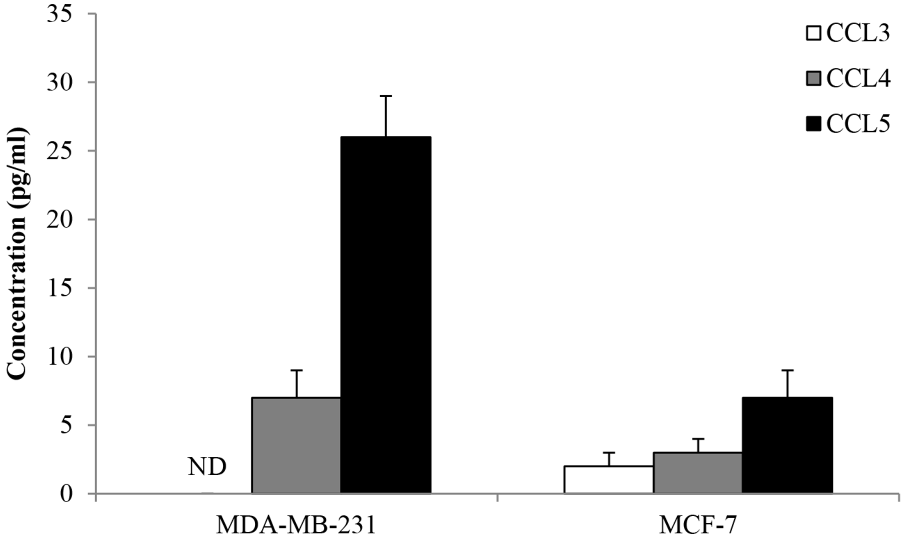

Supplementary Figure 26: The secretion of CCL3, CCL4 and CCL5 in breast cancer cells. 10^6 cells were cultured in serum-free fresh media for 24 h, and chemokine production was measured by ELISA. ND, not detected.

Supplementary Table 1: Correlation between CCL5 expression and clinical-phathological characteristics in breast cancer

Characteristics	Number	High CCL5	Low CCL5	P value
Age				P>0.05
≤45	11	8	3	
>45	17	14	3	
Tumor size				P>0.05
≤2 cm	16	12	4	
>2 cm	12	10	2	
Lymph node metastasis				P<0.05
Negative	10	5	5	
Positive	18	17	1	
Grade				P<0.05
1 or 2	9	6	3	
3	19	16	3	
Stage				P<0.05
I+II	14	9	5	
III	14	13	1	

Supplementary Table 2: Correlation between CCR5 expression and clinical-phathological characteristics in breast cancer

Characteristics	Number	High CCR5	Low CCR5	P value
Age				P>0.05
≤45	11	7	4	
>45	17	13	4	
Tumor size				P>0.05
≤2 cm	16	11	5	
>2 cm	12	9	3	
Lymph node metastasis				P<0.05
Negative	10	5	5	
Positive	18	15	3	
Grade				P>0.05
1 or 2	9	6	3	
3	19	14	5	
Stage				P>0.05
I+II	14	10	4	
III	14	10	4	

Supplementary Table 3: Primers for quantitative PCR

See Supplementary File 1
