# Supplementary material for: Lactate-activated macrophages induced aerobic glycolysis and epithelial-mesenchymal transition in breast cancer by regulation of CCL5-CCR5 axis: a positive metabolic feedback loop
Source: Oncotarget. 2017 Nov 30;8(66):110426–43. doi: 10.18632/oncotarget.22786 (PMC5746394; doi:10.18632/oncotarget.22786)
Supplement: Supplementary file 2 [file oncotarget-08-110426-s002.docx]

**[Table S3](http://www.sciencedirect.com/science/article/pii/S0304383516303676#ec0010)**

Supplementary Table 3. Primers for quantitative PCR

| Gene | Primer sequences |
| --- | --- |
| CCL2 | F: 5'- TCACCAGCAGCAAGTGTCCCA -3' |
|  | R: 5'- GGAGTGAGTGTTCAAGTCTTCGGA -3' |
| CCL3 | F: 5’-TGGTGACAACCGAGTGGCT-3′ |
|  | R:5’-TGGTGCCATGACTGCCTACA-3′ |
| CCL4 | F: 5′-GCTTCCTCGCAACTTTGTGG-3′ |
|  | R:5′-GCGGAGAGGAGTCCTGAGTA-3′ |
| CCL5 | F: 5'- CCGAAAGAACCGCCAAGTGTG -3' |
|  | R: 5'- CAAGAGCAAGCAGAAACAGGCAAAT -3' |
| CCL25 | F: 5'- CGATAAAACCGTCGCCCTACA -3' |
|  | R: 5'- AGGCCAACTCCCTCTTTCCAG -3' |
| CCL27 | F: 5'- AGTCTAGGCTGAGCAACATGAAGGG -3' |
|  | R: 5'- CAGTGCTGGGTGGCAGTAGGA -3' |
| CCL28 | F: 5'- ACTTGGCTGCTGTCATCCTTCAT -3' |
|  | R: 5'- TGCCCTGTTACTGTTCCTCTTGC -3' |
| CXCL10 | F: 5'- TGCATCAGCATTAGTAATCAACC -3' |
|  | R: 5'- TCAGACATCTCTTCTCACCCTTC -3' |
| CXCL12 | F: 5'- CGTGGTCCTTTGGGGTGAACTA -3' |
|  | R: 5'- ACTGATGGGGCAGCAATCCTCT -3' |
| CXCL16 | F: 5'- GAAACACCTGAGAGCTTACCATC -3' |
|  | R: 5'- AAGCATGTCCACATTCTTTGAGA -3' |
| DLL1 | F: 5'- GGGGTCATCCTTGTCCTCA-3' |
|  | R: 5'- GTTGTTCATGGTCTCCGTCTC -3' |
| DLL3 | F: 5'- CTCAACAACCTAAGGACGCA-3' |
|  | R: 5'- GATGGAAGGAGCAGATATGACA-3' |
| DLL4 | F: 5'- CTCCCTAGCTGTGGGTCAGA-3' |
|  | R: 5'- ACATAGTGGCCGAAGTGGTC -3' |
| Jagged1 | F: 5'- TGCTGCCTTTCAGTTTCGC-3' |
|  | R: 5'- AACGCCCGTGTTCTGCTTC-3' |
| Jagged2 | F: 5'- CAGGTGGACGGCTTTGAGT-3' |
|  | R: 5'- GTCGTTGACGTTGATATGGC-3' |
| Notch1 | F: 5'- GGCACTTTCTGTGAGGAGGA-3' |
|  | R: 5'- GCAGTCAGGCGTGTTGTTC-3' |
| Notch2 | F: 5'- TGCCCACAAGGCTACAAAG -3' |
|  | R: 5'- TCCATCTCACAACGAGGTCC-3' |
| Notch3 | F: 5'- TGATCGGCTCGGTAGTAATG-3' |
|  | R: 5'- CAACGCTCCCAGGTAGTCA-3' |
| TGF-β1 | F: 5'-CGCTAAGGCGAAAGCCCTCAATTT-3' |
|  | R: 5'-ACAGCAACAATTCCTGGCGATACC-3' |
| CCR5 | F: 5'- ACGGCATTGCTCCGTCTAAGTCAT-3' |
|  | R: 5'- ACCCTCCTTTGGCCACAGAGTAAA-3' |
| HK2 | F: 5'- TGGAGCGAGGTCTGAGCAA-3' |
|  | R: 5'- ACCAGCAGGACCCGGAAAT-3' |
| PKM2 | F: 5'- ATGTCGAAGCCCCATAGTGAA-3' |
|  | R: 5'- TGGGTGGTGAATCAATGTCCA-3' |
| LDHA | F: 5'- CAGCCCGATTCCGTTACCT-3' |
|  | R: 5'- CCAGCAACATTCATTCCACTC-3' |
| PGC-1α | F: 5'- GCTTTCTGGGTGGACTCAAC-3' |
|  | R: 5'- CTGCTAGCAAGTTTGCCTCA-3' |
| ERRα | F: 5'- GGGACTTGGAGAGCAAAGG -3' |
|  | R: 5'- CTGGAGTCTGCTTGGAGTTATT-3' |
| ATPsynth | F: 5'- TGCAAGGAACTTCCATGCCTC-3' |
|  | R: 5'- CGCCCAGTTTCTTCAAGATCAA-3' |
| CytC | F: 5'- CTTTGGGCGGAAGACAGGTC-3' |
|  | R: 5'- TTATTGGCGGCTGTGTAAGAG-3' |
| UCP1 | F: 5'- TCTCCACCAGGACAGTACAA -3' |
|  | R: 5'- CAGGATCCAAGTCGCAAGAA-3' |
| ACC | F: 5'- TGAGACTAGCCAAACAATCTCGT-3' |
|  | R: 5'- AGAAAGTAGAAGCTCCGATCCT-3' |
| FASN | F: 5'- AAGGACCTGTCTAGGTTTGATGC-3' |
|  | R: 5'- TGGCTTCATAGGTGACTTCCA-3' |
| Twist | F: 5'- CGGGAGTCCGCAGTCTTA-3' |
|  | R: 5'- TGAATCTTGCTCAGCTTGTC-3' |
| Snail | F: 5'- GAGGCGGTGGCAGACTAG-3' |
|  | R: 5'- GACACATCGGTCAGACCAG-3' |
| Slug | F: 5'- CTCTCTCCTCTTTCCGGATACT -3' |
|  | R: 5'- GCTTGGACTGTAGTCTTTCCTC-3' |
| 18S | F: 5'-GTAACCCGTTGAACCCCATT-3' |
|  | R: 5'-CCATCCAATGGGTAGTAGCG-3' |
